# *methylClass*: an R package to construct DNA methylation-based classification models

**DOI:** 10.1093/bib/bbad485

**Published:** 2024-01-09

**Authors:** Yu Liu

**Affiliations:** Laboratory of Pathology, Center for Cancer Research, National Cancer Institute, Bethesda, MD 20892, USA

**Keywords:** methylation, multi-omics, support vector machine, ensemble, feature selection, pan-cancer

## Abstract

DNA methylation profiling is a useful tool to increase the accuracy of a cancer diagnosis. However, a comprehensive R package specially for it is lacking. Hence, we developed the R package *methylClass* for methylation-based classification. Within it, we provide the eSVM (ensemble-based support vector machine) model to achieve much higher accuracy in methylation data classification than the popular random forest model and overcome the time-consuming problem of the traditional SVM. In addition, some novel feature selection methods are included in the package to improve the classification. Furthermore, because methylation data can be converted to other omics, such as copy number variation data, we also provide functions for multi-omics studies. The testing of this package on four datasets shows the accurate performance of our package, especially eSVM, which can be used in both methylation and multi-omics models and outperforms other methods in both cases. *methylClass* is available at: https://github.com/yuabrahamliu/methylClass.

## INTRODUCTION

Accurate pathological diagnosis is crucial for managing cancer patients, and many efforts have been made for it. In virtue of artificial intelligence (AI) techniques, various diagnostic classifiers emerge, such as the ones trained from hematoxylin and eosin images [1–3], stimulated Raman histology data [4], etc. Among them, the DNA methylation (DNAm) ones show outstanding performance [5–8].

When combining the Illumina Infinium Methylation array data with AI methods, several clinical-grade DNAm classifiers were created [5, 8], particularly suitable for individualized cancer diagnostics [9–12]. However, a comprehensive R package specially for it is lacking. Therefore, we developed the R package *methylClass* to fill in this gap.

Within it, various machine learning methods are covered, such as random forest (RF), support vector machine (SVM) and extreme gradient boosting (XGBoost). Among them, RF is fast and performs well, making it the most popular for DNAm classification.

On the other hand, if focusing on accuracy, SVM can outperform other methods. However, its time complexity prohibits applying it to large datasets, making SVM less popular. Hence, in this package, we develop a modified SVM method, eSVM (ensemble-based SVM), to achieve similar accuracy but take much less time, promoting the use of SVM-like classifiers.

Furthermore, we also include a multilayer perceptron neural network. However, when used on large datasets, it also shows a time-consuming problem, so we modified it to an eNeural (ensemble-based neural network) method, similar to eSVM.

In addition, the package provides some new feature selection and multi-omics integration methods. For example, the Single-Cell Manifold Preserving Feature Selection (*SCMER*) method can screen for markers preserving the manifold of original data [13], the *JVis* method can perform joint tSNE and uniform manifold approximation and projection (UMAP) embedding [14], and the multi-omics classification method Multi-Omics Graph cOnvolutional NETworks (*MOGONET*) can be used if other omics are available in addition to DNAm [15]. Similarly, eSVM and eNeural can also train multi-omics classifiers because of their internal ensemble framework. This is another advantage because they expand the application from SVM’s single-omic data to multi-omics data. These functions are tested with four cancer datasets here, including three methylation datasets and one multi-omics dataset.

## METHODS AND RESULTS

### Package overview

**Figure 1:**
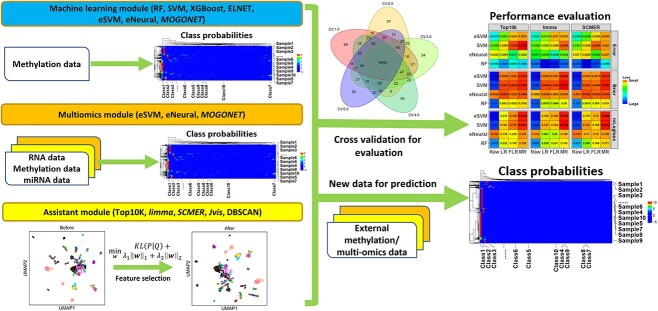
Package overview. The package contains three modules: the machine learning module, the multi-omics module and the assistant module. The classifiers trained calculate the posterior probabilities of samples belonging to different classes and determine the classification results accordingly, as shown by the heatmaps under the title “Class probabilities”. Their rows represent samples, and their columns represent classes. Another heatmap under the title “Performance evaluation” shows how the package evaluates different classifier performances after cross-validation. The labels on the left side show different model construction methods, the ones on the bottom represent different calibration methods, the top ones are the feature selection methods and the right ones are the loss metrics to evaluate the model performance. Hence, an entry in the heatmap indicates the loss of its model, which is trained based on the corresponding construction, calibration and feature selection methods.

The package has three modules (Figure 1). The first is a machine learning module. It constructs classifiers from DNAm data, including the methods of RF, SVM, XGBoost, ELNET (elastic net classification), eSVM, eNeural and *MOGONET*. The second module is a multi-omics module. It contains eSVM, eNeural and *MOGONET*. They can train a classifier not only from DNAm data but also from multi-omics data, including RNA, miRNA, copy number variation data, etc. The third module includes various assistant functions, such as feature selection, pan-cancer sample filtering, etc.

### eSVM uses less running time but achieves an accuracy as high as SVM

**Figure 2:**
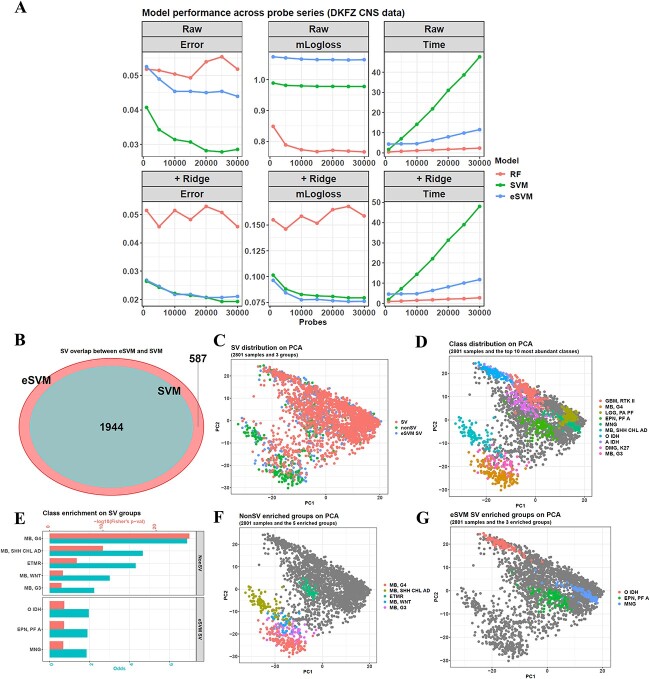
Comparison between SVM, eSVM and RF. (**A**) eSVM has similar accuracy to SVM when running on the DKFZ CNS data probe series but has a faster speed than SVM. Each dot in the plots represents one model. The *x*-axes indicate the probe numbers, and the *y*-axes indicate their misclassification error, mLogloss or running time (hours). The upper three facets show the performance of the raw eSVM, SVM and RF models, while the bottoms show the performance of their ridge-calibrated models. (**B**) In the DKFZ CNS dataset, the SVs of the eSVM model cover the SVs of the SVM model, explaining their accuracy similarity. (**C**) Distribution of the SVs, non-SVs and eSVM-specific SVs in the PCA embedding of DKFZ samples. (**D**) Distribution of the CNS methylation subclasses in the PCA embedding. There are 91 subclasses; the colored dots represent the top 10 most abundant ones, including GBM (RTK II) (Glioblastoma RTK II) (143 samples), MB (G4) (Medulloblastoma Group 4) (138 samples), LGG (PA PF) (Low-grade glioma pilocytic astrocytoma in posterior fossa) (114 samples), EPN (PF A) (Ependymoma posterior fossa A) (91 samples), MNG (Meningioma) (90 samples), MB (SHH CHL AD) (Medulloblastoma SHH child and adolescent) (84 samples), O IDH (Oligodendroglioma IDH) (80 samples), A IDH (Astrocytoma IDH) (78 samples), DMG (K27) (Pediatric diffuse midline gliomas H3 K27) (78 samples) and MB (G3) (Medulloblastoma Group 3) (77 samples). (**E**) Significantly enriched methylation classes can be found in the non-SVs (upper facet) and eSVM-specific SVs (bottom facet). The bars show the −log10 values of Fisher’s exact *P*-value and the odds of enrichment. Only the methylation classes with a Fisher's *P*-value < 0.05 are shown. (**F**) PCA embeddings of the five subclasses enriched in the non-SV group. They are MB (G4), MB (SHH CHL AD), ETMR (Embryonal tumor with multilayered rosettes), MB (WNT) (Medulloblastoma WNT) and MB (G3). (**G**) PCA embeddings of the three subclasses enriched in the eSVM-specific SV group. They are O IDH, EPN (PF A) and MNG.

We first tested three machine learning methods (RF, SVM and eSVM) on a central neural system (CNS) tumor dataset with 2801 samples and 91 classes (DKFZ CNS data, originally generated by German Cancer Research Center, Deutsches Krebsforschungszentrum, DKFZ) [5]. We performed a probe series experiment by selecting the top 1000, 5000, 10 000, until 30 000 most variable methylation probes from the data and then used them to construct different machine learning models. This was completed by calling the function *maincv* in the package. Then, the model performance was evaluated via a 5 by 5 nested cross-validation (CV), which was also fulfilled by *maincv* (Figure 2A). Finally, these model results were calibrated via ridge regression provided by the function *maincalibration*.

As described in Supplementary Data, eSVM was an ensemble model combining the traditional SVM model and the bagging framework and utilized the feature sampling step of bagging to relieve the time-consuming problem of SVM.

Given that the DNAm probes in the training data constructed a super-high dimensional feature space, making the samples there distributed very sparsely, a linear kernel was used for both SVM and eSVM because it could best separate sparse samples [16].

The result showed that both SVM and eSVM had a misclassification error less than RF. Before model calibration, almost all the probe numbers had a misclassification error on RF > 0.05, whereas eSVM had an average value of 0.0466, and that of SVM was 0.0317. After calibration, these two methods improved to around 0.02 (SVM average = 0.0219 and eSVM average = 0.0225), but RF only had an average of 0.0494. In addition, the ridge calibration also reduced the average mLogloss of SVM and eSVM to 0.0847 and 0.0807, whereas for RF, it was > 0.15 for most probe numbers. Hence, the advantage of support-vector-based methods was proved.

Although the accuracy of SVM and eSVM was similar, eSVM had a large advantage in running time. Among the three methods, RF was the most time-efficient. For a 5 by 5 nested CV running on 10 threads, if the probe number was 1000, the raw RF training only took 0.462 h, and as the probe number increased, the running time increased slightly until the largest value of 2.33 h for 30 000 probes. However, SVM showed a weaker performance here. For 1000 probes, it took 1.67 h to finish the nested CV, and when the probe number increased to 5000, its running time increased sharply to 6.98 h, which had been larger than the time of RF on 30 000 probes. This demonstrated the barrier to SVM being widely used. However, the running time of eSVM was much shorter. Although it needed 4.33 h for the initial 1000 probes, after that, it increased very slowly and took 4.46 h for 5000 probes, 4.52 h for 10 000 probes, until 11.5 h for 30 000 probes, much less than the SVM values of 6.98, 14.1 and 47.6 h.

For the calibration step, it was time-efficient. Ridge took an average of 0.464 h for the RF models, 0.31 h for the eSVM models and 0.332 h for the SVM models. Hence, the total time of raw model training plus calibration was similar to the raw one.

Next, we explored why eSVM had similar accuracy to SVM and compared their support vectors (SVs). If their SVs overlapped largely, the space margins they defined to separate the classes should be very close, leading to a similar accuracy. With the function *maintrain* in the package, we checked the models trained on the whole 2801 DKFZ samples, and for eSVM, we combined the SVs of its base learners, and the comparison showed that they covered all the 1944 SVs of the SVM model (Figure 2B). Hence, the margins of SVM and eSVM were similar.

Moreover, eSVM also owned 587 unique SVs, so for the total 2801 CNS samples in the dataset, 1944 were SVs shared by the two models, 587 were eSVM-specific SVs, whereas the remaining 270 were non-SVs for both models. Then, we checked the principle component analysis (PCA) embedding on the top 10 000 variable probes and found that the 2801 samples formed two clusters, and most of the 1944 SVs and the 587 eSVM-specific SVs were in the large cluster. In contrast, most of the 270 non-SVs were in the small cluster (Figure 2C). If looking at the cancer subclasses, most of them mixed in the large cluster (Figure 2D). This demonstrated that the samples in the large cluster were difficult to separate and so easy to get a penalty and became SVs. In contrast, the non-SVs were in the small cluster and were isolated from most samples in the large cluster, so they could be separated easily without penalty and became non-SVs.

We then checked what tumor subclasses were enriched in the SVs and non-SVs. For the 1944 SVs, no significantly enriched subclasses could be found. However, five subclasses were enriched in the non-SVs, including MB (G4), MB (SHH CHL AD), ETMR, MB (WNT) and MB (G3), and all of them belonged to the embryonal CNS tumor class (Figure 2E). Meanwhile, the eSVM-specific SVs were enriched in three subclasses of O IDH, EPN (PF A) and MNG. For their locations in the PCA embedding, four of the five non-SV subclasses were in the small PCA cluster, whereas all the three eSVM-specific SV subclasses were in the large cluster (Figure 2F and G).

### The top variable, *limma* and *SCMER* methods select largely different features

**Figure 3:**
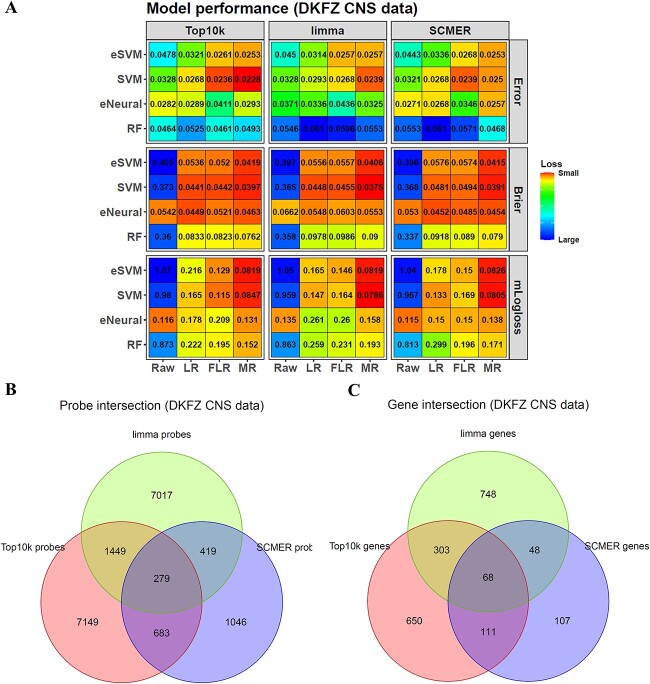
Influence of feature selection on model performance. (**A**) Different combinations of the raw models, calibration and feature selection methods show different performances. Each entry of the heatmap represents the metric value of one model. For the *x*-axis, Raw means no calibration, LR means calibration with logistic regression, FLR means calibration with Firth’s penalized logistic regression and MR means calibration with multinomial ridge regression. (**B**) Overlapping of the features selected by the three feature selection methods. (**C**) Overlapping of the genes matched by the three feature sets.

In addition to the model algorithms, the features used for classifier training were also important. To evaluate them on the DFKZ dataset, we used the function *mainfeature* in the package to select three kinds of features (DNAm probes here): the top 10 k most variable (top10k) features, *limma* features and *SCMER* features. Then, the functions *maincv* and *maincalibration* were used on them to train classifiers in the 5 by 5 CV framework. The result showed that the best five models were all SVM models combined with different features and calibration methods. In contrast, the 6–10th best models were all eSVM models (Figure 3A). The eNeural and RF models showed a weaker performance, but eNeural was better than RF. Among the three calibration methods, multinomial ridge regression (MR) was better than logistic regression (LR) and Firth’s logistic regression (FLR) because most top models used MR for calibration. For the feature selection methods, the top models covered all of them, and no one showed a significant advantage.

Among these features, *SCMER* features preserved the sample embedding, and they were largely different from the top10k and *limma* features because they needed to preserve the sample–sample similarity matrix first, making *SCMER* a graph-based method sensitive to sample changes. This was shown by the five outer training sets of the 5 by 5 nested CV. Although the sets had several different samples, they always had similar top10k or *limma* features, but not *SCMER* ones (Figure S1, see Supplementary Data). For the top10k features, each training set had 9560 same as others, whereas, for the *limma* features, 9164 out of 10 000 features were shared. However, for *SCMER*, only 2427 out of around 10 000 features were shared because the sample changes in each training set made the sample–sample similarity matrix change largely, and *SCMER* was sensitive to it.

We then compared the 9560, 9164 and 2427 common features of top10k, *limma* and *SCMER*. We found that they further shared only 279 features (Figure 3B), which were positively enriched in the OpenSea probe island region (Figure S2A, see Supplementary Data) and negatively enriched in the TSS1500 gene region (Figure S2B, see Supplementary Data).

In addition, we explored the functions of these probes by mapping the TSS200, TSS1500 and 1stExon probes to genes and checked their functions. The three feature groups shared 68 genes (Figure 3C). Functional enrichment on these shared genes and the unique ones of each feature group showed a close relationship to neural tumors (Figure S3, see Supplementary Data). More details could be found in Supplementary Data.

### The SVM/eSVM classifiers identify new subclasses in TCGA data

**Figure 4:**
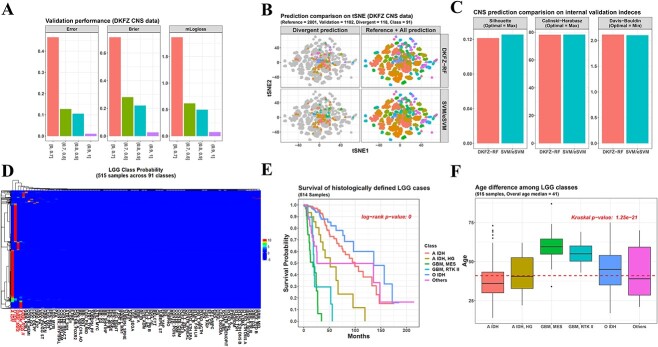
Application of SVM/eSVM on external CNS data. (**A**) Performance of aggregated labels of the SVM/eSVM-MR models on the DKFZ validation dataset. The samples are stratified into different groups according to their prediction confidence scores from the DKFZ RF classifier. The *x*-axes show the confidence score ranges of the groups, and their sample sizes are [0, 0.7] = 200, (0.7, 0.8] = 55, (0.8, 0.9] = 95 and (0.9, 1] = 752. (**B**) Among the 1104 DKFZ CNS validation samples, 2 have a DKFZ RF predicted label as NA and are removed from the analysis, and among the remaining 1102 validation samples, 118 are predicted by our SVM/eSVM model and the DKFZ RF model differently. The left column only colors the 118 differently predicted samples in the tSNE plot, whereas the right column colors all the reference (training) cohort and validation samples, with each color representing one CNS tumor subclass. (**C**) The three internal validation indices obtain their optimal value at our SVM/eSVM classification result. (**D**) The six SVM/eSVM-MR models jointly predict the methylation subclasses of the TCGA LGG dataset. From the aggregated label probability matrix, samples are mainly enriched in the subclasses of A IDH, O IDH, A IDH (HG) (Astrocytoma IDH high grade), GBM (MES) (Glioblastoma mesenchymal) and GBM (RTK II). The heatmap entries are the log2 transformed probability values followed by scaling along the heatmap row direction. The column names are the subclass names, and the five main ones are shown with a larger font size. (**E**) Among the 515 LGG samples, 514 have the survival data, and the log-rank *P*-value shows that different methylation subclasses have significantly different survival statuses. (**F**) Comparing the patient ages indicates that different methylation subclasses have significantly different ages in the LGG dataset.

So far, SVM and eSVM were the best machine learning models, and MR was the best calibration method. Hence, we trained the six SVM/eSVM-MR classifiers from the whole DKFZ dataset and applied them to another DKFZ validation set, including SVM-MR-top10k, SVM-MR-*limma*, SVM-MR-*SCMER*, eSVM-MR-top10k, eSVM-MR-*limma* and eSVM-MR-*SCMER* classifiers. Their prediction results were further aggregated to get a unified one, as described in Supplementary Data. Because the labels of the validation dataset were not the true labels but the predictions from the DKFZ RF classifier [5], to reduce the influence of the noise from these DKFZ predictions, we stratified the samples according to their prediction confidence from the DKFZ classifier and a group with higher confidence would have less noise in its labels. Correspondingly, the performance of our classifiers improved as the noise decreased (Figure 4A). For the samples with confidence > 0.9, assuming that all their labels were correct, the aggregated SVM/eSVM classifier obtained a misclassification error of 0.0106, a Brier score of 0.0263 and an mLogloss of 0.0733. As the label confidence decreased, the classifier performance was weakened.

Next, we compared all the predicted labels from our SVM/eSVM model with those from the DKFZ RF classifier. We found that 118 samples were predicted divergently by them (Figure 4B). Because no true label was available, we could not compare their accuracy. However, some clustering internal validation indices could be used to measure the aggregation of the clusters (actually classes here) and determine the optimal classification. We used three internal validation indices: the Silhouette, Calinski–Harabasz and Davis–Bouldin indices. The former two reached the optimal at their maximum values, whereas the third reached the optimal at the minimum value. We found that our SVM/eSVM classification had a larger Silhouette (0.126 versus 0.121) and Calinski–Harabasz index (78.281 versus 78.137) but a smaller Davis–Bouldin index (2.102 versus 2.115), demonstrating that our model was better because samples in the same class tended to have smaller distance with each other. In contrast, that in different classes tended to be farther away from each other (Figure 4C).

Because the current SVM/eSVM predictions were aggregated from the six SVM/eSVM classifiers, we also checked these indices for these six models separately. When comparing with DKFZ RF, all of their predictions had at least one index better than it. For the eSVM-MR-*limma*, SVM-MR-top10k and SVM-MR-*SCMER* models, all the three indices were better (eSVM-MR-*limma*: Silhouette = 0.126 > 0.121, Calinski = 78.180 > 78.137, Davis = 2.093 < 2.115; SVM-MR-top10k: Silhouette = 0.126, Calinski = 78.283, Davis = 2.100; SVM-MR-*SCMER*: Silhouette = 0.124, Calinski = 78.175, Davis = 2.113). For SVM-MR-*limma*, two of its three indices were better than DKFZ RF (Silhouette = 0.124, Davis = 2.111). For eSVM-MR-top10k and eSVM-MR-*SCMER*, one index was better (eSVM-MR-top10k: Davis = 2.074, eSVM-MR-*SCMER*: Davis = 2.091). Hence, the performance of these single classifiers also proved that the SVM/eSVM models were more accurate than DKFZ RF.

After this validation, we extended our classifiers to two The Cancer Genome Atlas (TCGA) CNS cancer datasets, low-grade glioma (LGG) and glioblastoma (GBM), to see their methylation subclasses because TCGA classified the samples using the histological system, but their labels from the methylation system were unknown. For the 515 LGG samples, the 6 SVM/eSVM-MR classifiers aggregately predicted them as 6 methylation subclasses, including A IDH, O IDH, GBM (MES), A IDH (HG), GBM (RTK II) and others. The “others” group was a combination of the samples belonging to subclasses with ≤ 10 samples (Figure 4D). Then, the survival data of the predicted subclasses were compared. A significant difference was detected (Figure 4E). The two largest subclasses, A IDH (220 samples) and O IDH (167 samples), showed the largest survival time with a median value of 28.8 and 23.2 months, whereas the two GBM subclasses GBM (MES) (36 samples) and GBM (RTK II) (20 samples) showed the shortest survival with a median of 12 and 17 months. Hence, although these two subclasses were diagnosed as LGG via TCGA’s histological system, their short survival status was more similar to GBM samples. If checking the patient ages, these two subclasses showed a much larger one than others (Figure 4F), consistent with the GBM situation that the patients always had an older age.

Next, to further validate our classifications of the GBM (MES) and GBM (RTK $\text{II}$) samples, we introduced the Sturm GBM subtyping system, which pathologists had already used as a reference to help in diagnosis [17]. The DNAm-based tSNE embedding showed that our GBM subtypes clustered together with the corresponding samples from the Sturm reference, confirming our predictions (Figure S4, see Supplementary Data). The details could be found in Supplementary Data.

For the TCGA GBM dataset, our classifiers identified three main subclasses, including GBM (RTK $\text{II}$), GBM (MES) and GBM (RTK I), all of which still belonged to GBM (Figure S5A, see Supplementary Data), and no significant difference was detected in their survival or patient age (Figure S5B and C, see Supplementary Data).

### The SVM/eSVM classifiers perform better than RF in sarcoma subclassification

**Figure 5:**
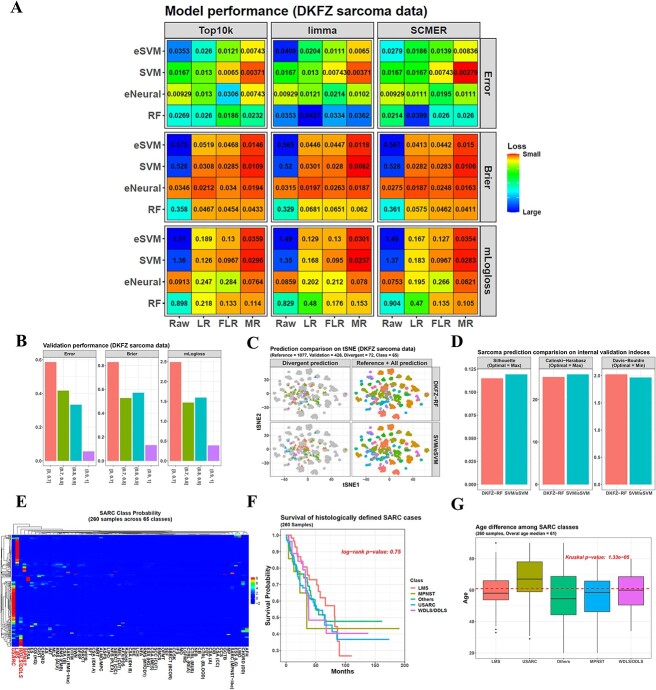
Application of SVM/eSVM on sarcoma data. (**A**) Different combinations of the raw models, calibration methods and features perform differently on the DKFZ sarcoma dataset in a 5 by 5 nested CV. (**B**) Performance of aggregated labels of the SVM/eSVM models on the sarcoma validation dataset. The samples are stratified into different groups according to their prediction confidence scores from the DKFZ RF classifier. The *x*-axes show the confidence score ranges of the groups, and their sample sizes are [0, 0.7] = 70, (0.7, 0.8] = 12, (0.8, 0.9] = 24 and (0.9, 1] = 322. (**C**) Among the 428 sarcoma validation samples, 72 are predicted differently by our SVM/eSVM model and the DKFZ RF model. The left column only colors the 72 differently predicted samples in the tSNE plot, while the right column colors all the reference (training) cohort and validation samples, with each color representing one sarcoma subclass. (**D**) All the three internal validation indices obtain their optimal value at our SVM/eSVM classification. (**E**) The six SVM/eSVM-MR models jointly predict the methylation subclasses of the TCGA sarcoma dataset. From the aggregated label probability matrix, samples are mainly enriched in the subclasses of USARC (Undifferentiated sarcoma), LMS (Leiomyosarcoma), WDLS/DDLS (Well-differentiated liposarcoma/dedifferentiated liposarcoma) and MPNST (Malignant peripheral nerve sheath tumor). The heatmap entries are the log2 transformed probability values followed by scaling along the heatmap row direction. The column names are the subclass names, and the four main ones are shown with a larger font size. (**F**) Survival analysis shows no significant difference exists among the sarcoma subclasses. (**G**) Age comparison finds that USARC patients have a significantly older age than others.

We also tested our package on the DKFZ sarcoma dataset with 1077 samples and 65 classes [8], and the combination of different raw models, calibration methods and features showed different performances in a 5 by 5 nested CV (Figure 5A). SVM/eSVM still performed the best, then eNeural and finally RF. For the calibration methods, MR was better than others. For the features, *SCMER* achieved the smallest misclassification error of 0.00279 when combined with SVM and MR.

Next, we trained the six SVM/eSVM-MR classifiers from the whole DKFZ sarcoma dataset and applied them to another DKFZ sarcoma validation set with 428 samples. Their prediction results were aggregated together to get a unified one. Because the labels of this validation dataset were also the predictions from the DKFZ RF classifier rather than the true labels [8], the previous stratification method was used to reduce the influence of the wrong predictions from the DKFZ RF classifier. For the sample group with the largest confidence score > 0.9, the aggregated labels from our classifiers obtained an error of 0.0559. As the confidence score decreased, the divergence between our classifiers and the DKFZ one increased (Figure 5B).

When comparing our model predictions with the DKFZ RF model labels, 72 out of the 428 validation samples were predicted differently (Figure 5C). Then, the three internal validation indices were used again to measure the classifications. The result showed that our classification was better than DKFZ, with both Silhouette and Calinski–Harabasz indices larger (0.119 versus 0.115 and 25.936 versus 25.309), and Davis–Bouldin index smaller (1.966 versus 2.023) (Figure 5D).

When checking the indices for the six single SVM/eSVM models separately, they all had three indices better than DKFZ RF (eSVM-MR-top10k: Silhouette = 0.122, Calinski = 25.924, Davies = 1.943; SVM-MR-top10k: Silhouette = 0.121, Calinski = 25.930, Davies = 1.950; eSVM-MR-*SCMER*: Silhouette = 0.117, Calinski = 25.692, Davies = 1.98; eSVM-MR-*limma*: Silhouette = 0.120, Calinski = 25.836, Davies = 1.981; SVM-MR-*limma*: Silhouette = 0.120, Calinski = 25.802, Davies = 1.981; SVM-MR-*SCMER*: Silhouette = 0.118, Calinski = 25.713, Davies = 1.989). It demonstrated the better performance of our SVM/eSVM models.

Next, we extended the six SVM/eSVM-MR classifiers to the TCGA SARC dataset containing 260 sarcoma samples and explored their methylation subclasses. After the aggregation of the six classifiers, their combined label distribution matrix showed that the samples mainly belonged to four subclasses, including USARC (93 samples), LMS (60 samples), WDLS/DDLS (27 samples) and MPNST (14 samples). Other small classes with ≤ 10 samples were combined (a total of 66 samples) (Figure 5E). The survival analysis did not show a significant difference among them (Figure 5F). However, a significant difference was found in the patient ages because the USARC patients were much older than others (Figure 5G). This was consistent with the reports that USARC frequently occurs in senior people [18].

### eSVM outperforms *MOGONET* in multi-omics data classification

In addition to DNAm data, eSVM could also train models from multi-omics data, which was an advantage over SVM. We tested this using 1064 TCGA breast invasive carcinoma (BRCA) samples covering three omics, i.e. 450K/27K DNAm, RNA-seq and miRNA-seq. We chose this dataset because we wanted to compare the performance of eSVM with another multi-omics classifier named *MOGONET*, and the TCGA BRCA dataset was used in its original study to test its performance [15].

*MOGONET* was a graph convolutional network (GCN) specially developed for multi-omics data and was reported as better than other methods. It trained one GCN model for each omic, and then, a fully connected neural network was used to perform aggregation on all the GCN results and their interaction terms. We collected it into our package so that it could be called directly by the function *maincv* or *maintrain*. However, we added some modifications to it. The most important one was the aggregation step. Instead of aggregating on both the original GCN results and their interaction terms, we would discard the interaction terms if a dataset had a class number > 2. This was because many cancer datasets had a large class number due to the cancer heterogenicity, such as the DKFZ CNS and DKFZ sarcoma (91 and 65 classes), and their interaction term number could be > 10 000, forming a large time complexity for the aggregation step. On the other hand, to compensate for the performance impairment led by this interaction removal, we increased the base learner number so that each omic could be assigned > 1 GCN base learner, as described in Supplementary Data.

In the original *MOGONET* study with interaction terms, all its testing datasets had a small class number, such as the BRCA dataset here. It contained five PAM50 subtypes (Luminal A, Luminal B, basal-like, *HER2*-enriched and normal-like), so the interaction number was only 125. Hence, in addition to our modified *MOGONET* model, we could also use the original *MOGONET* with interaction terms. Moreover, we completely followed the original *MOGONET* study to select the features for classifier training, mainly based on ANOVA.

**Figure 6:**
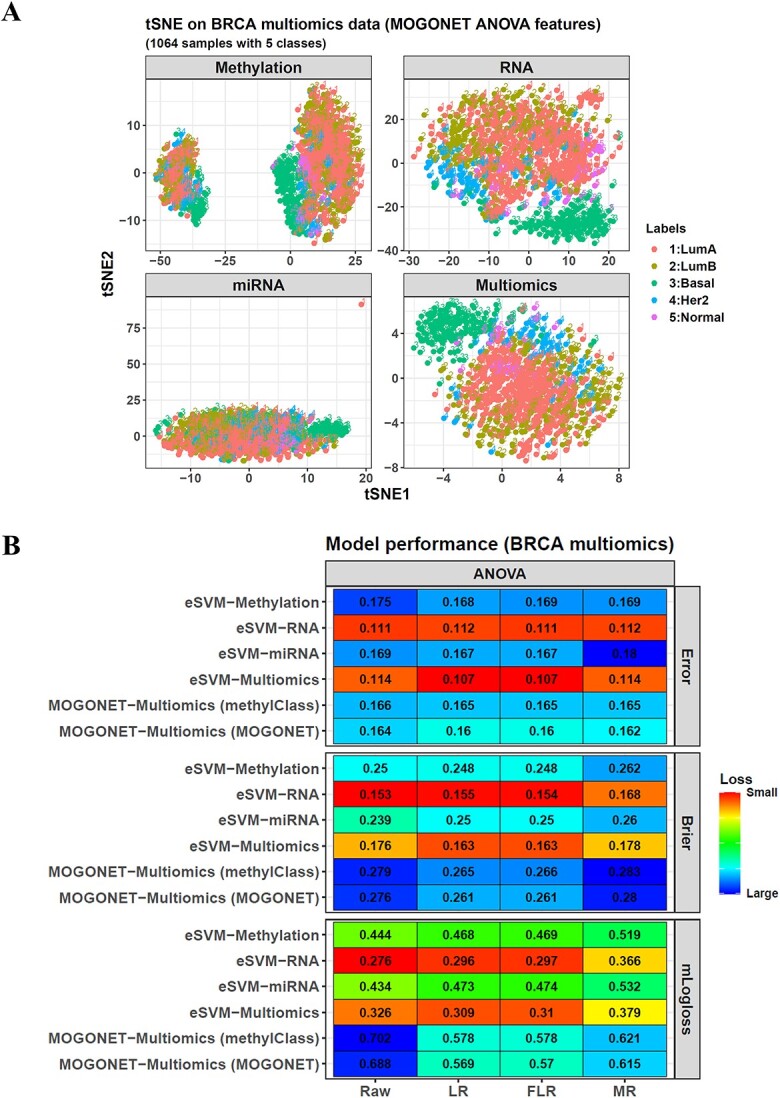
Application of the package on multi-omics data. (**A**) The package incorporates the JVis method to conduct tSNE embedding not only on each single-omic data but also on all the omics via integrating their sample adjacency. (**B**) The performance of eSVM, the modified MOGONET and the original MOGONET on the BRCA multi-omics dataset. The features used to construct the classifiers are the ANOVA-based features generated following the original MOGONET study.

Next, we checked the data embedding using the function *mainjvisR* in the package, which followed the *JVis* joint embedding method [14]. It generated the tSNE plots, not only for the three omics individually but also for the joint embedding integrating the sample-sample adjacency of all the omics (Figure 6A). The embedding demonstrated the difficulty of this classification because the five BRCA classes were always mixed.

Correspondingly, all the classifiers had a misclassification error > 0.1 in the 5 by 5 nested CV (Figure 6B), but the performance of eSVM was much better than *MOGONET*, even if all the features were selected following the *MOGONET* ANOVA method. The best two models were eSVM-LR and eSVM-FLR. Their error rates were both 0.107. In contrast, the error rates of all *MOGONET* models were ≥ 0.16. This performance was weaker than eSVM but matched their original study results (error = 0.171 in a normal 5-fold CV).

Notably, the original *MOGONET* always showed a smaller error than the modified one, illustrating the advantage of calculating the interaction terms for aggregation. Although we introduced more base learners to the modified model, it did not completely compensate for the loss.

Moreover, we also used eSVM on the individual omic data and found that the eSVM-RNA models performed better than the eSVM-DNAm and eSVM-miRNA ones. Their best error rate was 0.111. However, this was still weaker than the eSVM multi-omics model.

Meanwhile, another study also used this TCGA BRCA dataset to test its classifier, *meth-SemiCancer* [19]. However, it only used the DNAm part because *meth-SemiCancer* was a single-omic method to predict cancer subtypes from DNAm data. Its uniqueness was that it was a semi-supervised classifier. We were interested in it and used the TCGA BRCA data to build *meth-SemiCancer*. Then, we compared its performance with our DNAm models. The result showed that SVM and eSVM were more accurate than *meth-SemiCancer* (Figure S6, see Supplementary Data). The details could be found in Supplementary Data.

### SVM/eSVM classify pan-cancer samples accurately

We next tried our models on a pan-cancer dataset. We randomly selected 1712 pan-cancer samples from 34 public datasets, all from the Infinium MethylationEPIC platform and covering 37 cancer types.

Although these samples had clear histological labels, because the histological system and the methylation system were different, the histological label of a sample might not match its methylation cluster. Hence, as described in Supplementary Data, we performed a filtering process and only kept the samples with a matching relationship between their histological labels and methylation clusters.

We first used *mainfeature* to select the dataset’s top10k, *limma* and *SCMER* probe features. Then, we combined these features to get their union. After that, *mainjvisR* embedded the samples based on this union. From the tSNE result, most samples with the same histological labels tended to cluster together. However, some samples were diffused into clusters dominated by a different histological label, reflecting the divergence between the histological and methylation systems (Figure S7A, see Supplementary Data).

Then, we used the functions *clustergrid* and *labelclusters* on this tSNE embedding and filtered out samples without matching between their histological labels and DNAm clusters. Finally, 1198 of the 1712 samples passed the filtering process. They covered 33 histological pan-cancer classes (Figure S7B, see Supplementary Data). The details were in Supplementary Data.

**Figure 7:**
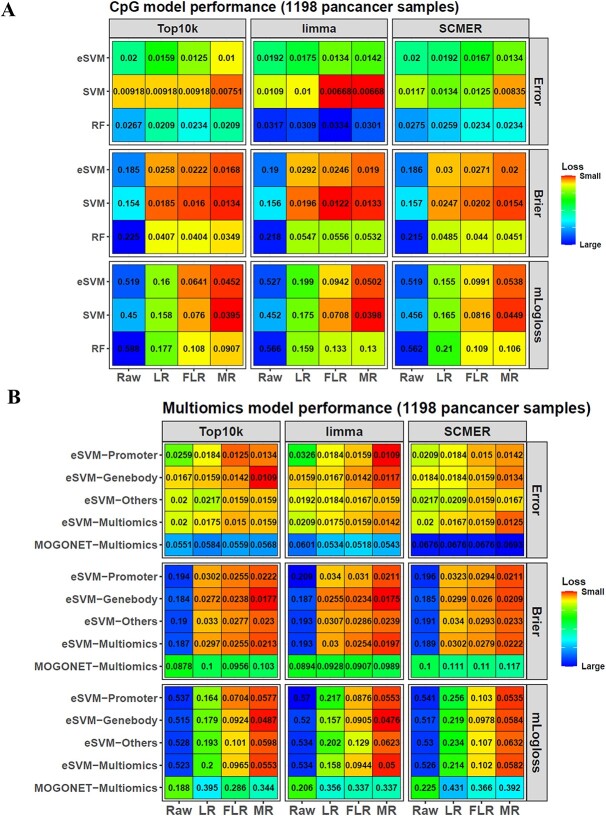
Application of the package on pan-cancer data. (**A**) Different models show different classification performances on the pan-cancer DNAm probe data. (**B**) With the multi-omics strategy of eSVM, the methylation probe data can be used to train a multi-omics model.

Next, the functions *maincv* and *maincalibration* were used on these samples to construct pan-cancer classifiers with their top10k, *limma* and *SCMER* features, respectively. The 5 by 5 CV results showed that the SVM/eSVM models classified the pan-cancer samples much better than the RF model. When coupling with FLR/MR calibration and *limma* features, SVM reached the smallest error rate (0.00668) of all the models (Figure 7A). Meanwhile, the best error rate of eSVM models was 0.01, whereas that of RF was 0.0209.

Moreover, we also constructed multi-omics classifiers on this dataset with eSVM and *MOGONET* (modified *MOGONET* without interaction terms). It was fulfilled by splitting the original data probes into three groups: gene promoter probes, gene body probes and other probes. Then, each group was treated as a single omic so that multi-omics models could be built on these pseudo-multi-omics data, and the top10k, *limma* and *SCMER* features could also be selected from each pseudo-omic. The tSNE embedding from *mainjvisR* showed that the pan-cancer classes could be separated well across all the pseudo-omics and the feature selection methods (Figure S8, see Supplementary Data), and the classification result showed that the eSVM-MR-*SCMER* model reached the best error of 0.0125 among all the multi-omics classifiers (Figure 7B). In contrast, for the *MOGONET* models, their best error was only 0.0518. Hence, eSVM still had a large advantage over *MOGONET*.

However, if applying eSVM to the individual omic data, the eSVM-MR-*limma* model on the promoter probe data and the eSVM-MR-top10k model on the gene body probe data could reach an error of 0.0109, better than the eSVM multi-omics models. The reason might be that the divergence among these single-omic models was small, violating the requirement of divergence when ensembling them into the multi-omics model, so the consistent error of the single-omic classifiers weakened the final ensemble.

Finally, we noted another study using the traditional SVM model to classify pan-cancer data, but the DNAm beta values of this dataset were from the RRBS (reduced-representation bisulfite sequencing) platform [20]. We checked the performance of our models on the same RRBS dataset, and the result showed that, in addition to DNAm microarray, our models could also be applied to RRBS data (Figure S9, see Supplementary Data). The details could be found in Supplementary Data.

## DISCUSSION

DNAm profiling is a useful tool for tumor diagnosis [5–8], and large margin classifiers, such as SVM, have better accuracy than other methods to classify DNAm data. However, SVM has a time-consuming problem because its sequential minimal optimization (SMO) solving algorithm will largely slow down when many SVs are in the solution. Hence, we developed eSVM to relieve this problem. It shortens the sample vector ${\boldsymbol{x}}_{\boldsymbol{i}}$ via its feature sampling step during bagging and so reduces the time complexity of the inner product calculation on ${\boldsymbol{x}}_{\boldsymbol{i}}^{\boldsymbol{T}}{\boldsymbol{x}}_{\boldsymbol{j}}$, which SMO needs. Hence, eSVM is less time-consuming than SVM.

In addition, eSVM also has another advantage: the expanded application to multi-omics data, which has promising power in cancer diagnosis, because cancer prognostication is a multi-modal problem that is driven by markers in histology, clinical data and genomics [1–8]. DNAm-based classifiers only capture part of the oncogenesis multi-omics network, and combined with other omics, they can get more information and increase accuracy.

Therefore, it is understandable that multi-omics models have better accuracy than DNAm ones, and not only multi-omics but other single-omic models, such as RNA ones, may outperform DNAm models. In our BRCA case study, in addition to the multi-omics eSVM models, other RNA-based eSVM models also showed higher accuracy than the DNAm ones. Because this result is from only one dataset, we cannot conclude that the advantage of RNA is universal, but if it were, the reason might be that the RNA transcriptional changes are more dynamic and highly correlated to the protein level and so have a more direct relationship with the cancer phenotype.

However, in practical cases, getting multi-omics data may be difficult, and even for the RNA data alone, they are not easy to obtain because RNA profiling is dependent on fresh tumor tissues and RNA is unstable [21]. In contrast, DNAm profiling can be reliably performed on formalin-fixed and paraffin-embedded tissues, and DNAm microarray always has high data quality [22]. This makes DNAm classification easy to implement. Hence, improving the DNAm single-omic classifier is necessary.

Although we have shown the contribution of eSVM in this area, other directions need to be explored, such as alternative calibration methods. This study includes the LR, FLR and MR methods. They are derived from the popular multi-response linear regression concept, aiming to predict each of the multi-classes separately from their raw predictions. Although this concept has been widely used, other algorithms, such as neural networks and the supra Bayesian procedure, may work better and are worth trying.

Based on these AI techniques, our package has shown excellent efficacy in overcoming the inter-observer variability of histopathological diagnosis, which poses a big challenge to the neural tumor case study, largely increasing the misclassification rate [10, 23, 24]. This problem is even more severe for the sarcoma case because approximately half of the sarcoma entities lack morphologic or molecular hallmarks [25]. However, DNAm classification solves it and improves the diagnostic precision, which can be seen from our classifiers’ high accuracy. Furthermore, our SVM/eSVM classifier has also shown an ability to revise initial histological diagnosis. It identified several GBM samples from the histological TCGA LGG dataset, and the following analysis supported this result because these samples showed strong GBM characteristics. This diagnostic change has a profound impact because it resulted in a change in the samples’ WHO (World Health Organization) grading from LGG’s lower grade (stage II or III) to GBM’s higher one (stage IV) [26].

In addition, the DNAm classifiers can continue to be reinforced because of the rapid accumulation of DNAm data to train them. For the DNAm data type, it is not restricted to microarray data; DNAm sequencing data can also be used with our package. For the sample type, it is not limited to postoperative tumor samples; cell-free DNA (cfDNA) methylation samples are another potential usage because cfDNA released by a tumor also carries its genomic and epigenetic properties and can be extracted non-invasively from body fluid [27, 28].

In summary, we provide the package *methylClass* for various DNAm classification tasks.

**Key Points**

- *methylClass* is an R package for methylation-based classification.
- eSVM overcomes the time-consuming problem of traditional SVM.
- eSVM outperforms other algorithms in both methylation and multi-omics classification.

## Supplementary Material

SupplementaryData_R_bbad485

tutorial_methylClass_bbad485

## Notes

**Yu Liu** PhD, is a Research Fellow (VP) at Laboratory of Pathology, Center for Cancer Research, National Cancer Institute.

## References

[1] Jin L, Shi F, Chun Q, et al. Artificial intelligence neuropathologist for glioma classification using deep learning on hematoxylin and eosin stained slide images and molecular markers. Neuro Oncol 2020;23:44–52. 10.1093/neuonc/noaa163 PMC7850049 32663285

[2] Lu MY, Williamson DFK, Chen TY, et al. Data-efficient and weakly supervised computational pathology on whole-slide images. Nat Biomed Eng 2021;5:555–70. 33649564 10.1038/s41551-020-00682-w PMC8711640

[3] Lu MY, Chen TY, Williamson DFK, et al. AI-based pathology predicts origins for cancers of unknown primary. Nature 2021;594:106–10. 33953404 10.1038/s41586-021-03512-4

[4] Hollon TC, Pandian B, Adapa AR, et al. Near real-time intraoperative brain tumor diagnosis using stimulated Raman histology and deep neural networks. Nat Med 2020;26:52–8. 31907460 10.1038/s41591-019-0715-9 PMC6960329

[5] Capper D, Jones DTW, Sill M, et al. DNA methylation-based classification of central nervous system tumours. Nature 2018;555:469–74. 29539639 10.1038/nature26000 PMC6093218

[6] Zhang J, Fan J, Wang P, et al. Construction of diagnostic and subtyping models for renal cell carcinoma by genome-wide DNA methylation profiles. Transl Androl Urol 2021;10:4161–72. 34984182 10.21037/tau-21-674 PMC8661251

[7] Zheng C, Xu R. Predicting cancer origins with a DNA methylation-based deep neural network model. PloS One 2020;15:e0226461. 32384093 10.1371/journal.pone.0226461 PMC7209244

[8] Koelsche C, Schrimpf D, Stichel D, et al. Sarcoma classification by DNA methylation profiling. Nat Commun 2021;12:498. 33479225 10.1038/s41467-020-20603-4 PMC7819999

[9] Pajtler Kristian W, Witt H, Sill M, et al. Molecular classification of ependymal tumors across all CNS compartments, histopathological grades, and age groups. Cancer Cell 2015;27:728–43. 25965575 10.1016/j.ccell.2015.04.002 PMC4712639

[10] Sturm D, Orr Brent A, Toprak Umut H, et al. New brain tumor entities emerge from molecular classification of CNS-PNETs. Cell 2016;164:1060–72. 26919435 10.1016/j.cell.2016.01.015 PMC5139621

[11] Sahm F, Schrimpf D, Stichel D, et al. DNA methylation-based classification and grading system for meningioma: a multicentre, retrospective analysis. Lancet Oncol 2017;18:682–94. 28314689 10.1016/S1470-2045(17)30155-9

[12] Reinhardt A, Stichel D, Schrimpf D, et al. Anaplastic astrocytoma with piloid features, a novel molecular class of IDH wildtype glioma with recurrent MAPK pathway, CDKN2A/B and ATRX alterations. Acta Neuropathol 2018;136:273–91. 29564591 10.1007/s00401-018-1837-8

[13] Liang S, Mohanty V, Dou J, et al. Single-cell manifold-preserving feature selection for detecting rare cell populations. Nat Comput Sci 2021;1:374–84. 36969355 10.1038/s43588-021-00070-7 PMC10035340

[14] Do VH, Canzar S. A generalization of t-SNE and UMAP to single-cell multimodal omics. Genome Biol 2021;22:130. 33941244 10.1186/s13059-021-02356-5 PMC8091681

[15] Wang T, Shao W, Huang Z, et al. MOGONET integrates multi-omics data using graph convolutional networks allowing patient classification and biomarker identification. Nat Commun 2021;12:3445. 34103512 10.1038/s41467-021-23774-w PMC8187432

[16] Maros ME, Capper D, Jones DTW, et al. Machine learning workflows to estimate class probabilities for precision cancer diagnostics on DNA methylation microarray data. Nat Protoc 2020;15:479–512. 31932775 10.1038/s41596-019-0251-6

[17] Sturm D, Witt H, Hovestadt V, et al. Hotspot mutations in H3F3A and IDH1 define distinct epigenetic and biological subgroups of glioblastoma. Cancer Cell 2012;22:425–37. 23079654 10.1016/j.ccr.2012.08.024

[18] Parikh RC, Lorenzo M, Hess LM, et al. Treatment patterns and survival among older adults in the United States with advanced soft-tissue sarcomas. Clin Sarcoma Res 2018;8:8. 29744029 10.1186/s13569-018-0094-x PMC5932822

[19] Choi JM, Park C, Chae H. meth-SemiCancer: a cancer subtype classification framework via semi-supervised learning utilizing DNA methylation profiles. BMC Bioinformatics 2023;24:168. 37101254 10.1186/s12859-023-05272-6 PMC10131478

[20] Zhang S, He S, Zhu X, et al. DNA methylation profiling to determine the primary sites of metastatic cancers using formalin-fixed paraffin-embedded tissues. Nat Commun 2023;14:5686. 37709764 10.1038/s41467-023-41015-0 PMC10502058

[21] Khan J, Wei JS, Ringnér M, et al. Classification and diagnostic prediction of cancers using gene expression profiling and artificial neural networks. Nat Med 2001;7:673–9. 11385503 10.1038/89044 PMC1282521

[22] Hovestadt V, Remke M, Kool M, et al. Robust molecular subgrouping and copy-number profiling of medulloblastoma from small amounts of archival tumour material using high-density DNA methylation arrays. Acta Neuropathol 2013;125:913–6. 23670100 10.1007/s00401-013-1126-5 PMC3661908

[23] van den Bent MJ . Interobserver variation of the histopathological diagnosis in clinical trials on glioma: a clinician’s perspective. Acta Neuropathol 2010;120:297–304. 20644945 10.1007/s00401-010-0725-7 PMC2910894

[24] Ellison DW, Kocak M, Figarella-Branger D, et al. Histopathological grading of pediatric ependymoma: reproducibility and clinical relevance in European trial cohorts. J Negat Results Biomed 2011;10:7. 21627842 10.1186/1477-5751-10-7 PMC3117833

[25] Gatta G, van der Zwan JM, Casali PG, et al. Rare cancers are not so rare: the rare cancer burden in Europe. Eur J Cancer 2011;47:2493–511. 22033323 10.1016/j.ejca.2011.08.008

[26] Louis DN, Ohgaki H, Wiestler OD, et al. The 2007 WHO classification of tumours of the central nervous system. Acta Neuropathol 2007;114:97–109. 17618441 10.1007/s00401-007-0243-4 PMC1929165

[27] Heitzer E, Haque IS, Roberts CES, Speicher MR. Current and future perspectives of liquid biopsies in genomics-driven oncology. Nat Rev Genet 2019;20:71–88. 30410101 10.1038/s41576-018-0071-5

[28] Cristiano S, Leal A, Phallen J, et al. Genome-wide cell-free DNA fragmentation in patients with cancer. Nature 2019;570:385–9. 31142840 10.1038/s41586-019-1272-6 PMC6774252

