# Supplementary material for: methylClass: an R package to construct DNA methylation-based classification models
Source: Brief Bioinform. 2024 Jan 9;25(1):bbad485. doi: 10.1093/bib/bbad485 (PMC10782803; doi:10.1093/bib/bbad485)
Supplement: SupplementaryData_R_bbad485 [file supplementarydata_r_bbad485.pdf]

Supplementary Data

Contents

Supplementary Figures 2
Supplementary Results..12
Features for neural tumor classification show a functional relationship with this disease 12
The Sturm system confirms the GBM samples predicted by the SVM/eSVM model..............13
eSVM outperforms *meth-SemiCancer* 13
The *methylClass* package can also be applied to DNAm sequencing data...............................15
Supplementary Methods 16
Data collection and preprocessing...16
Feature selection..20
Cross-validation assignment 22
Machine learning model training 22
Model calibration and performance evaluation..31
Internal validation indices calculation..33
Prediction of new samples..33
DNA methylation probe distribution analysis..34
Gene functional enrichment analysis 34
Multi-omics data visualization..35
Pan-cancer data filtering..37
References...41

Supplementary Figures

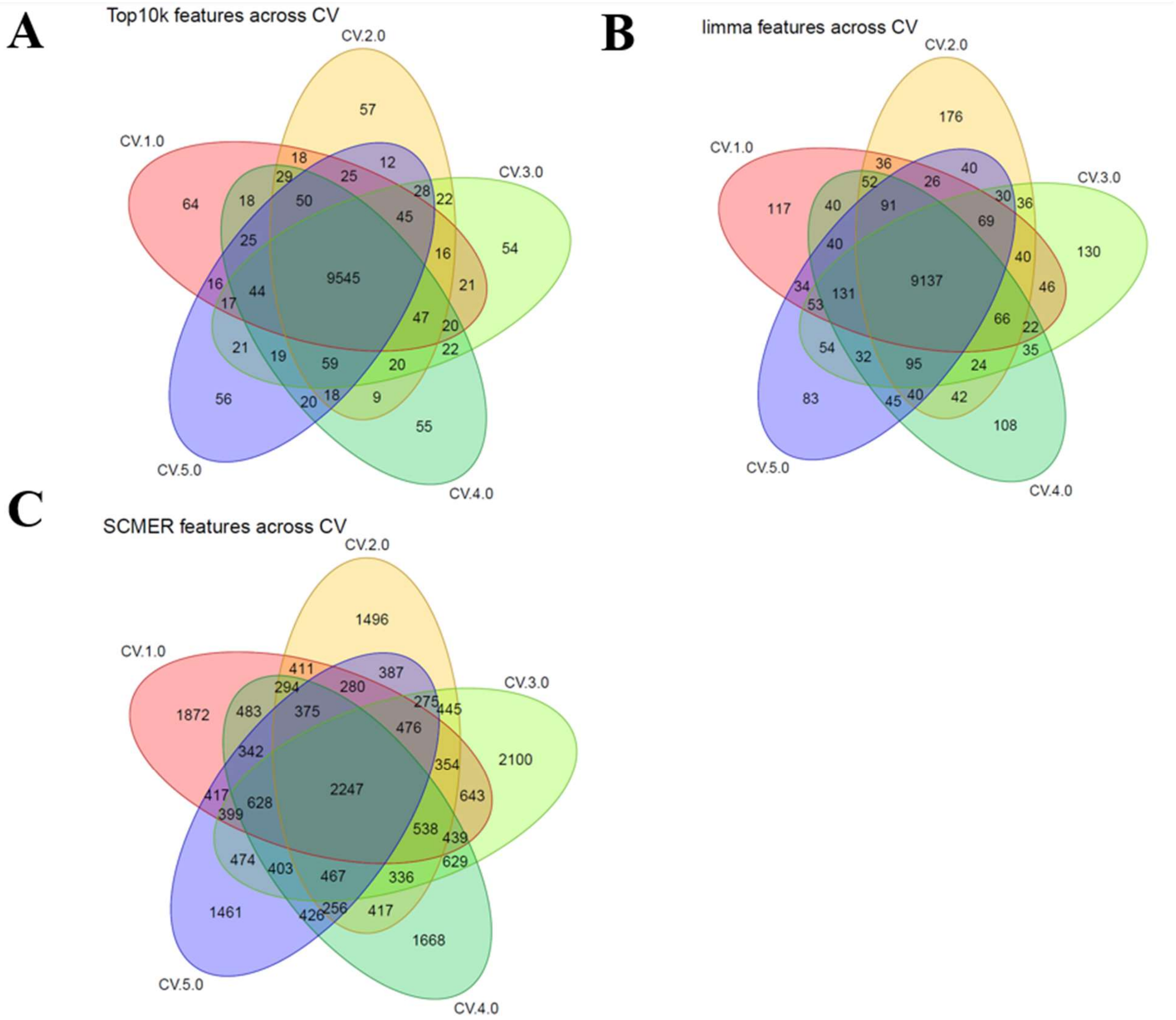

Fig. S1. Selected feature overlapping among different cross-validation loops. (A) and (B) For the top10k and *limma* methods, their selected features do not change largely among different training sets of the 5 outer CV (cross-validation) loops, as shown by a shared feature number of 9560 and 9164 out of 10000, respectively. (C) *SCMER*'s feature selection is sensitive to the sample changes of different training sets, as shown by a shared feature number as low as 2427 out of around 10000 features from each training set.

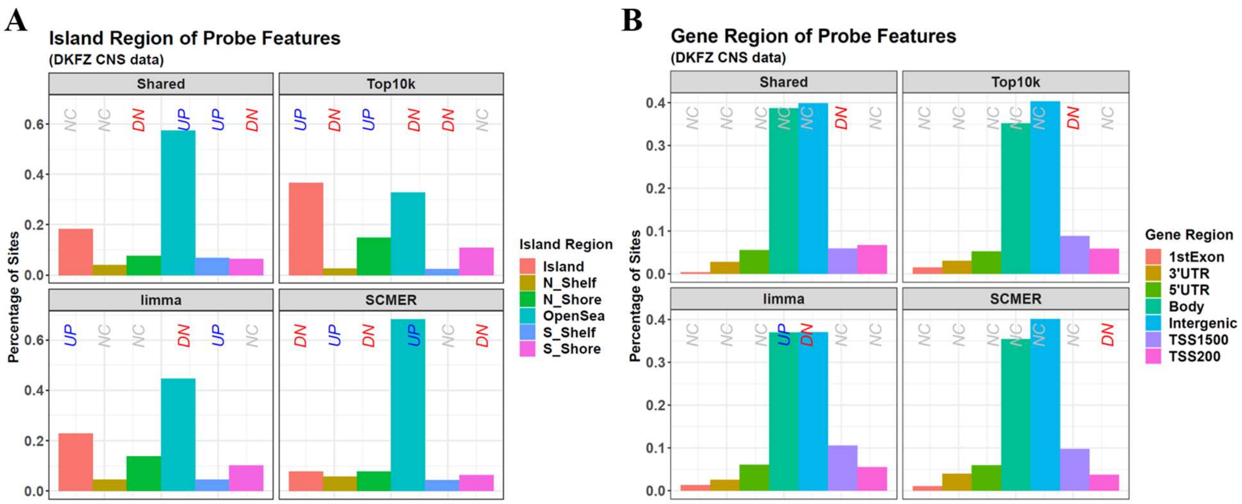

Fig. S2. Genomic region distribution of the methylation probes selected by different methods. (A) Island region distribution of the probes from different feature sets, including the shared probes of the top10k, *limma*, *SCMER* sets, and their unique ones. (B) Gene region distribution of the probes from different feature sets, including the shared probes of the 3 sets and their unique ones. The text labels "UP", "DN", and "NC" indicate that the probes are positively, negatively, and not significantly enriched in each region.

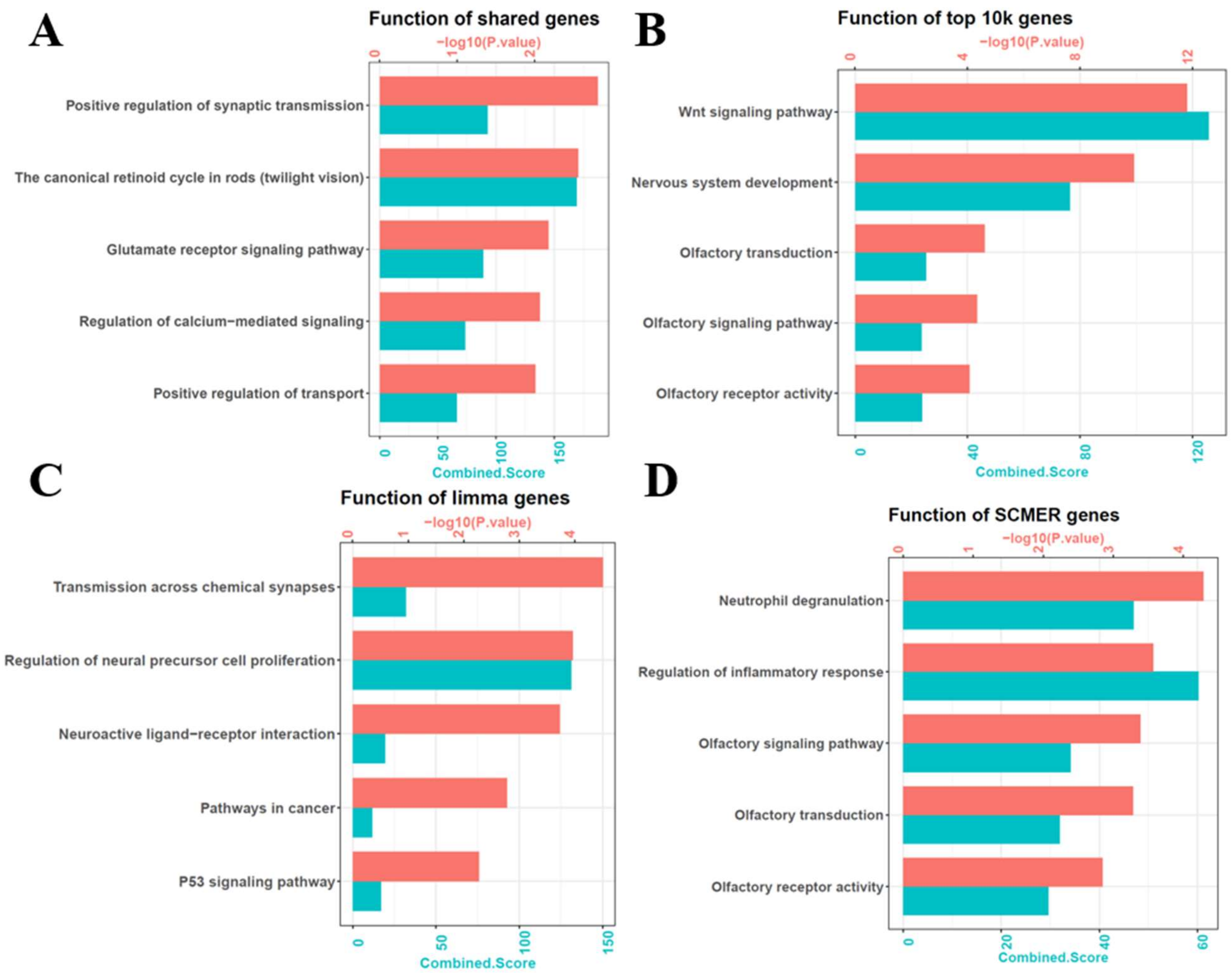

Fig. S3. The functional enrichment results of genes from different feature sets. (A) Results of the genes shared by the top10k, *limma*, and *SCMER* feature sets. (B) to (D) are results from the uniquely mapped genes of the 3 probe sets, respectively. The red bars indicate their functional enrichment p-values (-log10 transformed) and cyan bars indicate combined scores calculated by *EnrichR*.

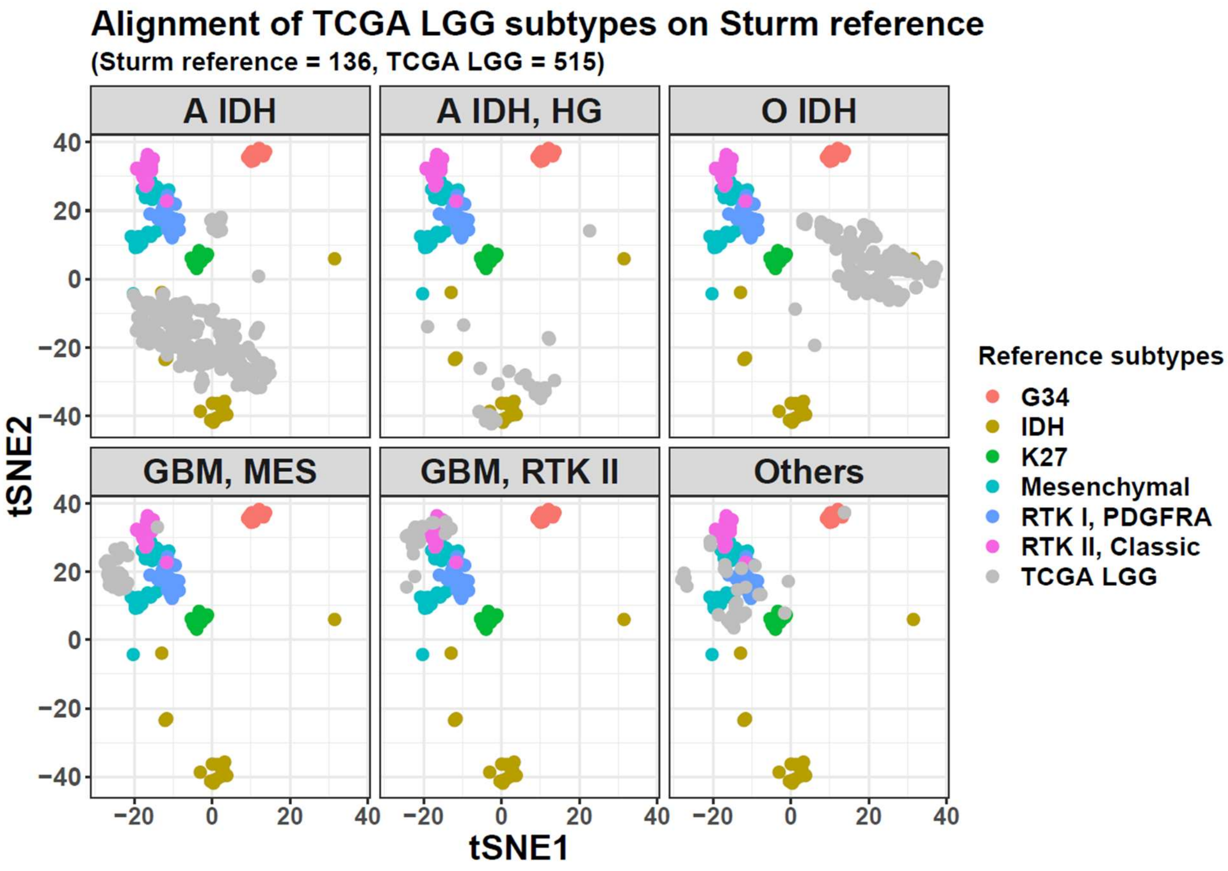

Fig. S4. Alignment of TCGA LGG samples on Sturm reference. The colorful dots represent samples from the original Sturm study belonging to its 6 GBM subtypes, including G34, IDH, K27, Mesenchymal, RTK I (PDGFRA), and RTK II (Classic). The grey dots are samples from the TCGA LGG dataset. All the facets show the same tSNE embedding space and contain the same Sturm samples, but each includes a different TCGA sample subtype predicted by our *methylClass* package. The facet titles show the TCGA subtype names. It is noteworthy that because this Sturm system was published in 2012, and at that time, IDH samples were assigned under the GBM class, but now, they are considered a separate entity from GBM, so although the Sturm system is called a GBM subtyping system, its IDH samples are not GBM ones.

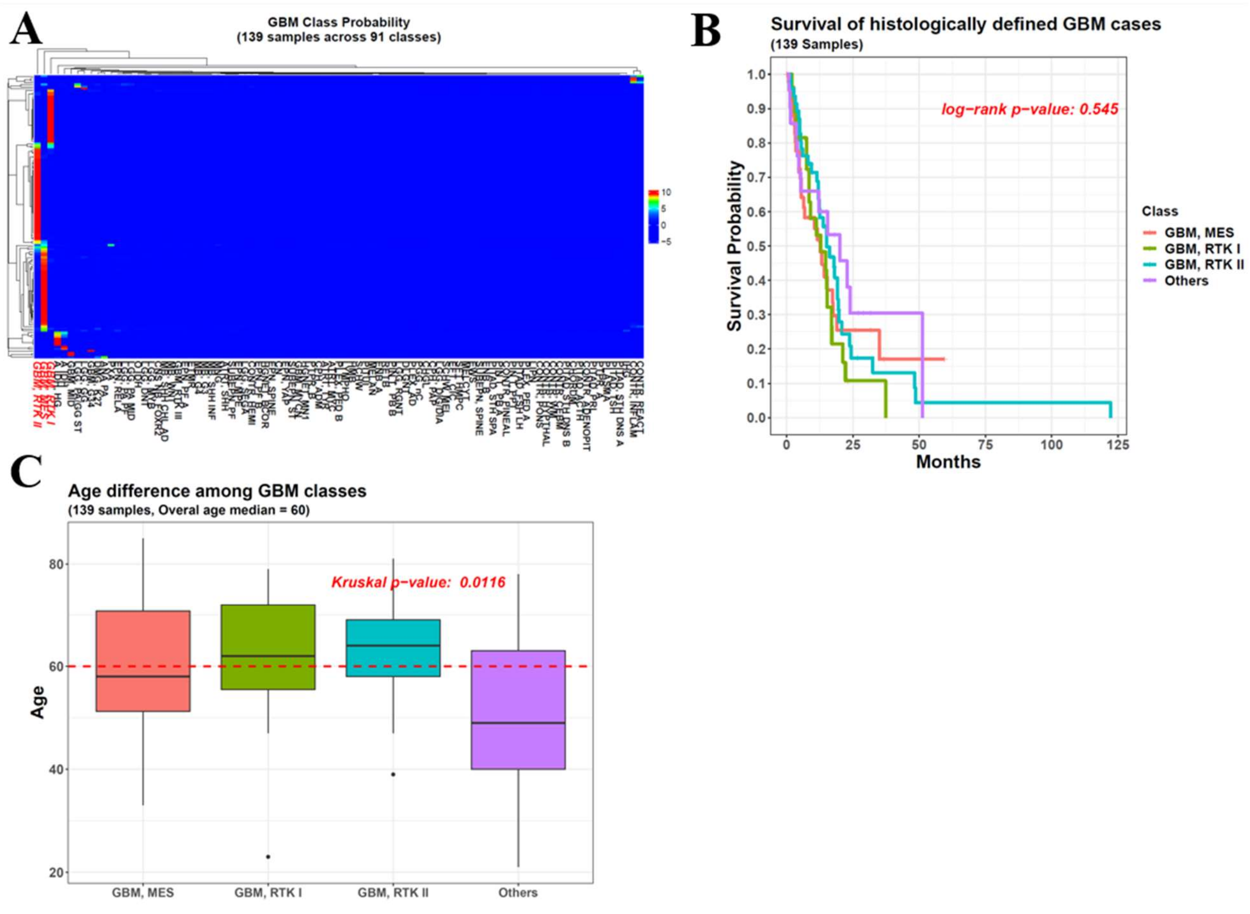

Fig. S5. Application of SVM/eSVM on TCGA GBM data. (A) The 6 SVM/eSVM-MR models jointly predict the methylation subclasses of the 139 450K GBM samples. From the aggregated label probability matrix, samples are mainly enriched in the subclasses of GBM (RTK II) (Glioblastoma RTK II), GBM (MES) (Glioblastoma mesenchymal), and GBM (RTK I) (Glioblastoma RTK I). The color of the entries indicates the log2 transformed probability values followed by scaling along the heatmap row direction. The column names are the subclass names, and the 3 main ones are shown with red color and a larger font size. (B) The median survival time is 9.3 months for the whole GBM dataset. (C) The GBM (MES), GBM (RTK I), and GBM (RTK II) samples are older than others.

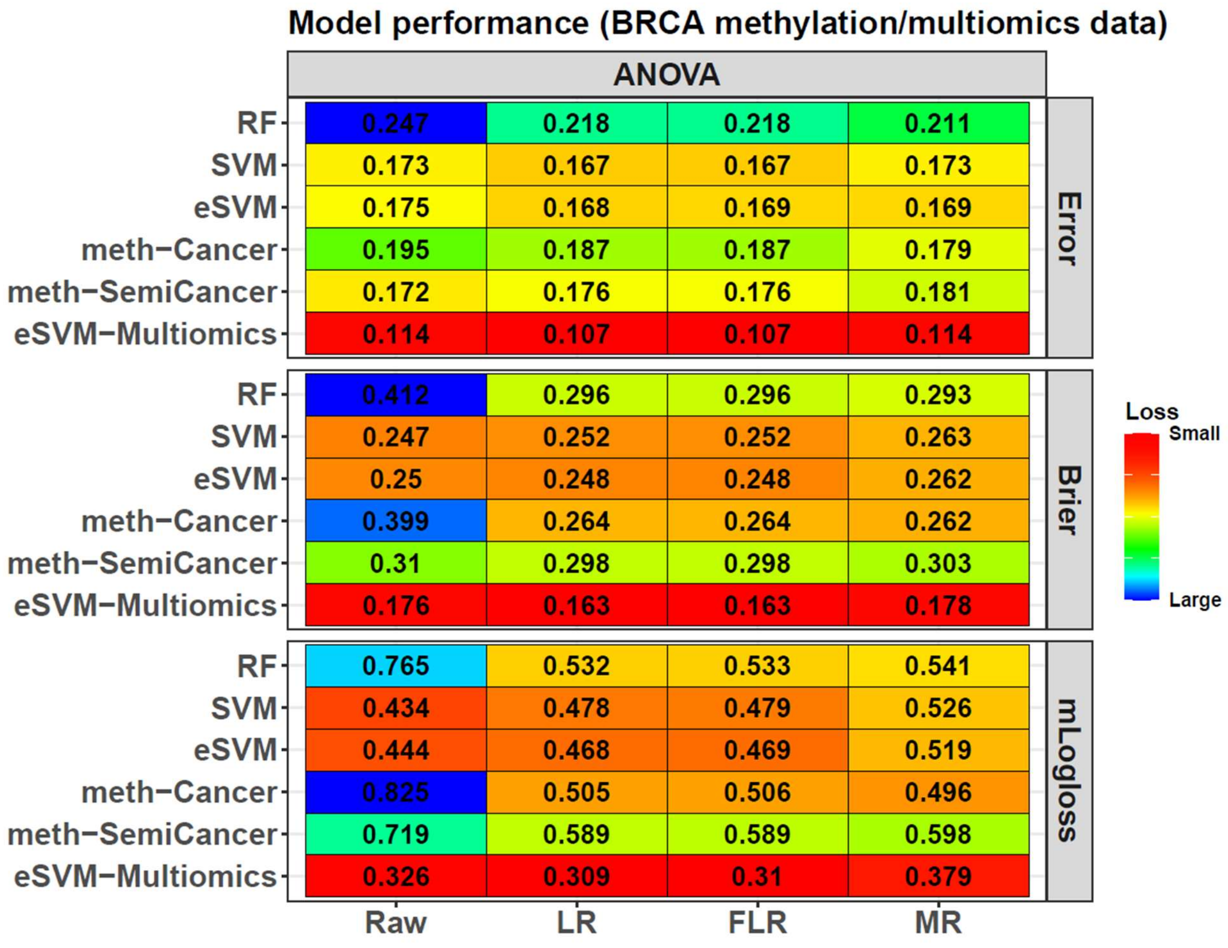

Fig. S6. The performance of various models on the TCGA BRCA dataset. Among them, the RF, SVM, and eSVM models are from our *methylClass* package, and the *meth-Cancer* and *meth-SemiCancer* models are from another study.

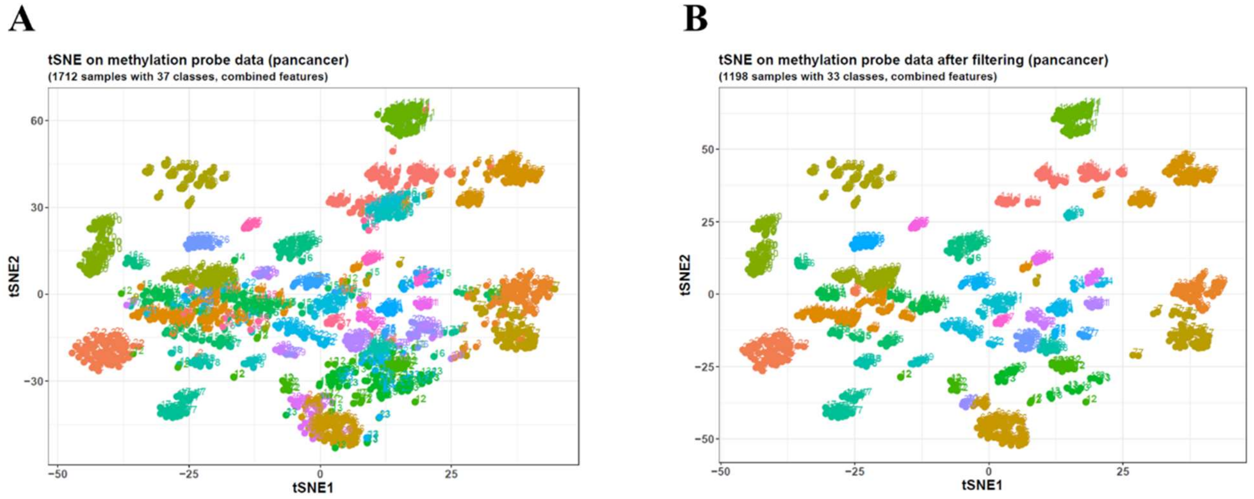

Fig. S7. Filtering on pan-cancer samples. (A) After the top10k, *limma*, and *SCMER* feature selection, *mainjvisR* can embed the 1712 pan-cancer samples based on the union of these feature probes. The result tSNE plot shows that some samples diffuse into the DNA methylation (DNAm) clusters dominated by different histological labels, indicating the difference between the DNAm and the histological systems. (B) After filtering by the *clustergrid* function, the matching relationship between the pan-cancer histological labels and the DNAm clusters becomes clear. For the index numbers of the dots in (A) and (B), they represent different cancer types, including 1) Acute myelogenous leukemia; 2) High-grade serous adenocarcinoma; 3) Malignant rhabdoid tumor; 4) Osteosarcoma; 5) T-lymphoblastic leukemia/lymphoma; 6) Colorectal adenoma; 7) Atypical teratoid/rhabdoid tumor; 8) Meningioma; 9) Chordoma; 10) Pituitary adenoma; 11) B-acute lymphoblastic leukemia; 12) Lung adenocarcinoma; 13) Intraductal tubulopapillary neoplasm; 14) Myxofibrosarcoma; 15) Malignant peripheral nerve sheath tumor; 16) Neuroendocrine tumor; 17) Rhabdomyosarcoma; 18) Breast adenocarcinoma; 19) Squamous cell carcinoma; 20) Acute lymphoblastic leukemia/lymphoma; 21) Chondrosarcoma; 22) Leiomyosarcoma; 23) Invasive cholangiocarcinoma; 24) Endometrial stromal sarcoma; 25) Ewing's sarcoma; 26) Gastrointestinal stromal tumor; 27) Adrenal adenoma; 28) Acral lentiginous

melanoma; 29) Adrenocortical carcinoma; 30) Colorectal adenocarcinoma; 31) Paraganglioma/pheochromocytoma; 32) Solitary fibrous tumor; 33) Clear cell sarcoma (kidney); 34) Langerhans cell histiocytosis; 35) Papillay carcinoma; 36) Anaplastic thyroid carcinoma; 37) Chondroblastoma.

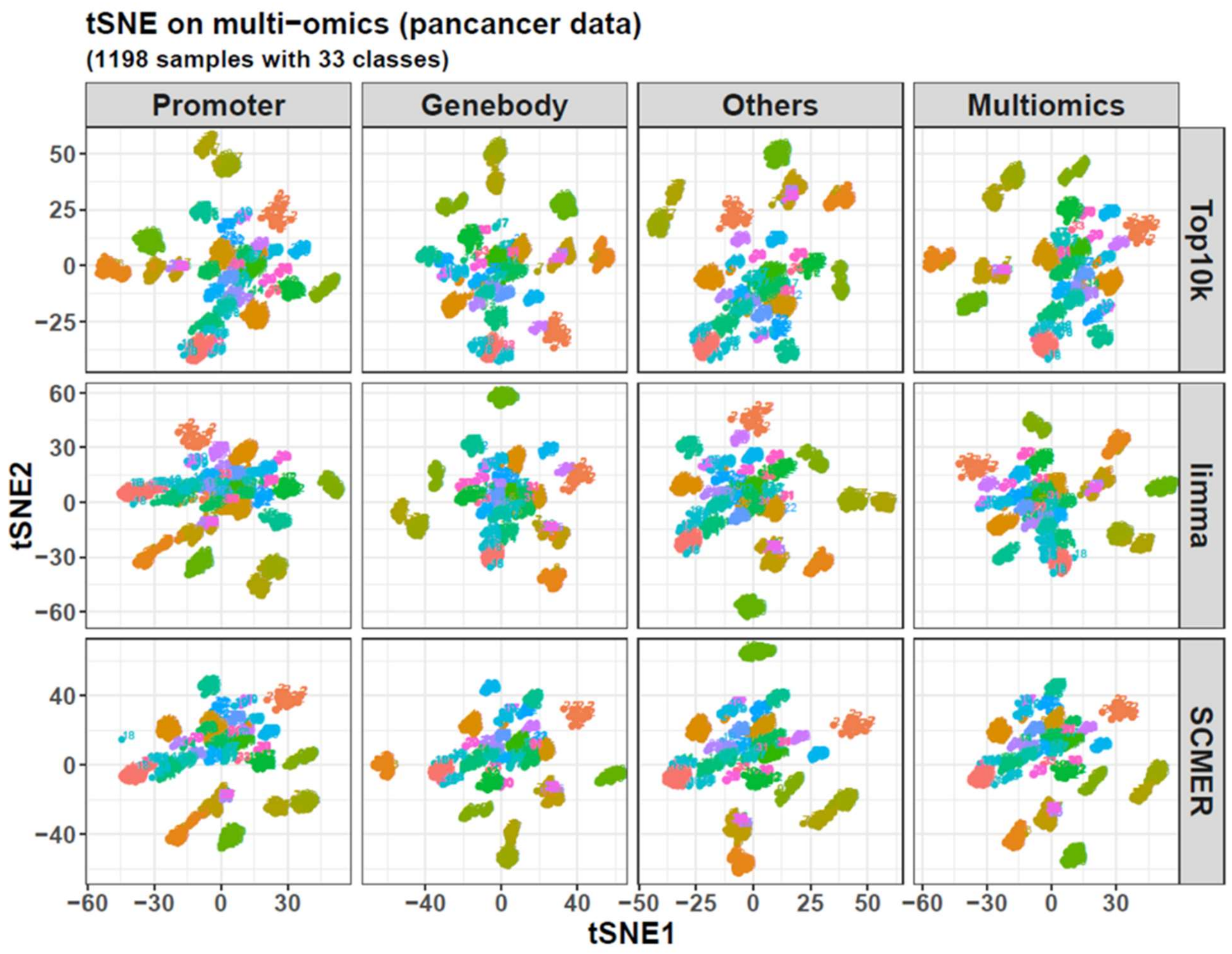

Fig. S8. Pseudo-multi-omics pan-cancer data embedding. The pan-cancer probe data can be converted to pseudo-multi-omics data by splitting the DNAm probes into 3 groups: gene promoter probes, gene body probes, and other probes, with each group corresponding to one omic. After different feature selection steps, *mainjvisR* can embed the samples based on single-omic or multi-omics data.

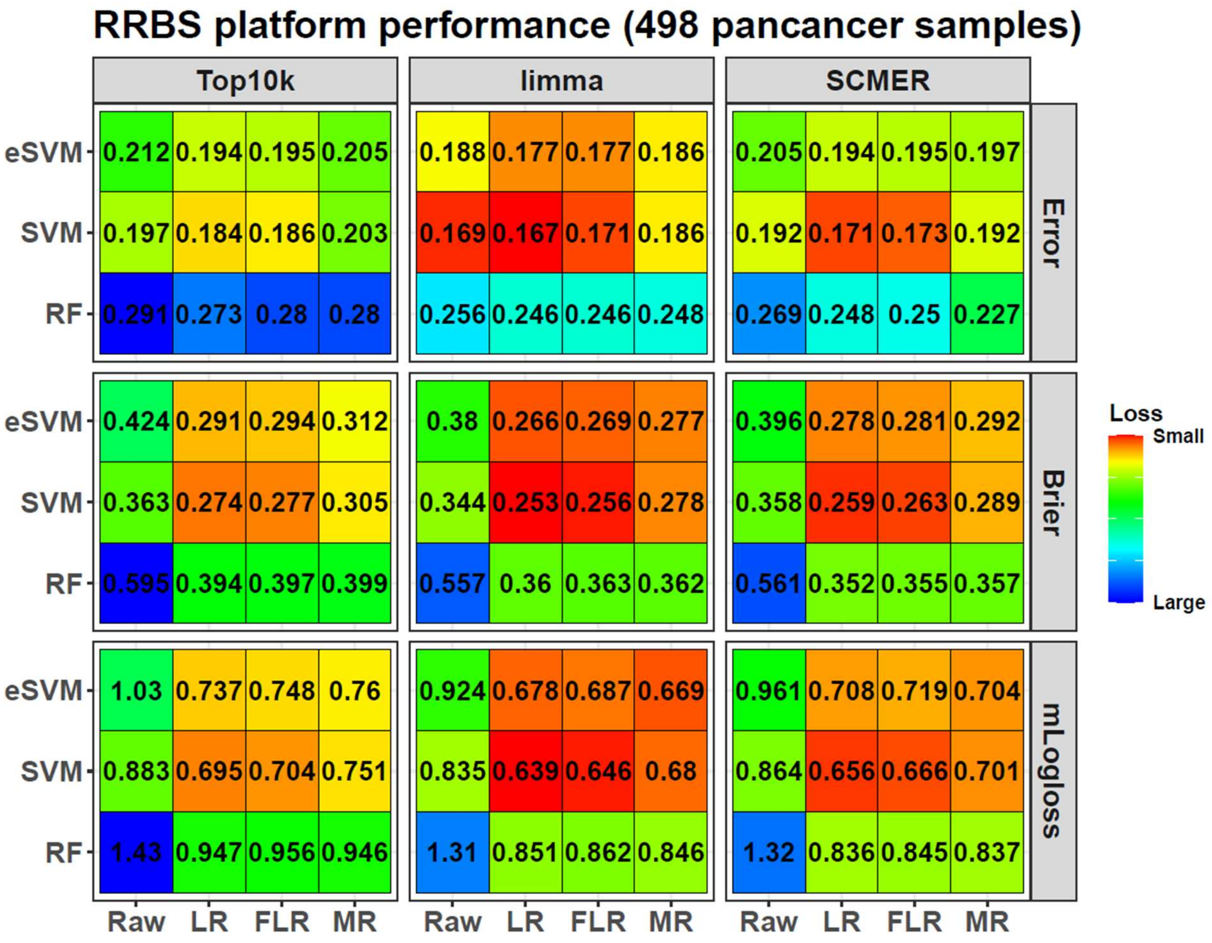

Fig. S9. Application of the package on RRBS data. Different models show different classification performances on the pan-cancer RRBS data.

Supplementary Results

Features for neural tumor classification show a functional relationship with this disease

After mapping the DNAm probes in the top10k, *limma*, and *SCMER* feature sets to genes, we checked the functional enrichment of their shared and unique genes, respectively. The 3 feature sets shared 68 genes (Fig. 3C), which showed a close relationship to the neural system because of their enriched functions, such as "Positive regulation of synaptic transmission" and "Glutamate receptor signaling pathway" (Fig. S3A). This was understandable, given that the training dataset was a neural tumor one. Furthermore, it was consistent with previous reports that altered glutamate secretion in neural tumors promoted their progression [1, 2], such as glioblastoma (GBM). It could secrete a considerable amount of glutamate, causing excitotoxicity in normal neurons and increasing expressions of glutamate transporters and glutamine synthetase, which then recruited tumor-associated microglia/macrophage (TAM), an important mediator of tumor cell survival [3, 4].

In addition to these shared genes' functions, the 3 feature groups also had their unique genes, and the *limma*-specific ones showed a close association with cancer-relevant processes such as "Pathways in cancer" and "P53 signaling pathway" (Fig. S3C). Indeed, previous studies had indicated that P53 mutation was one of the most frequent in brain tumors, and almost 50% of the glioma samples had such a mutation [5], which could promote the disease program and affect treatment strategies [6, 7]. In addition, P53 promoter methylation was found in more than 20% of primary GBM tumors [8].

On the other hand, the top10k and *SCMER* unique groups were enriched in the functions of "Olfactory transduction", "Olfactory signaling pathway" and "Olfactory receptor activity" (Fig.

S3B and D). Existing reports showed that olfactory receptors (ORs) were expressed in many cancers, including neural tumors [9-13]. Their somatic mutations were detected in > 60% of glioma samples, and OR5D18 was the most frequently mutated one. For other OR genes, survival analysis showed that OR51E1 expression was a biomarker of unfavorable survival in glioma, and OR2C1 expression was favorable [12]. In GBM, DNA methylation comparison indicated that OR51F2 was a potential downstream effector for its progression [13]. These reports validated the functional enrichment results here.

The Sturm system confirms the GBM samples predicted by the SVM/eSVM model

To further prove that our classifications of the GBM (MES) and GBM (RTK II) samples were correct, we introduced the Sturm system, which defined 6 GBM subgroups based on global DNAm patterns and had already been used by pathologists as a reference to help in diagnosis [14]. We downloaded the DNAm profiles of the original Sturm study and embedded them and the TCGA LGG samples into the same tSNE space. Then, we checked whether our predicted TCGA subtypes could cluster with the corresponding ones of the Sturm reference. It was clear from the tSNE result that our GBM (MES) samples were located very close to the corresponding Mesenchymal cluster in the Sturm system, and our GBM (RTK II) samples mixed with Sturm's RTK II (Classic) cluster (Fig. S4). Hence, the Sturm system confirmed our prediction on the GBM samples. In addition, our A IDH, A IDH (HG), and O IDH samples were located in Sturm's IDH cluster area, so it was reasonable to predict these TCGA LGG samples as IDH. It should be noted that because this Sturm study was published in 2012, and at that time, IDH samples were assigned under the GBM class, but now, they are considered a separate entity from GBM, so although the Sturm system is called a GBM subtyping system, its IDH samples are not GBM ones [14, 15].

eSVM outperforms *meth-SemiCancer*

The neural network classifier *meth-SemiCancer* was a single-omic method to predict cancer subtypes from DNAm data [16]. In its original study, the DNAm part of the TCGA BRCA dataset was used to test its performance. Its uniqueness was that it was a semi-supervised classifier using both labeled and unlabeled DNAm profiles. It first pre-trained on the TCGA BRCA samples with subtype labels. Then, it accepted many unlabeled BRCA samples from various GEO (Gene Expression Omnibus) datasets and used the pre-trained model to predict their pseudo-labels. Finally, a classifier was generated from all the labeled and pseudo-labeled samples. Like other semi-supervised methods, it utilized the data distribution information from the unlabeled samples to enhance the classifier's accuracy. Since we already had the TCGA BRCA data, it was convenient to build *meth-SemiCancer*. Hence, we collected the GEO unlabeled DNAm datasets used in its original study and used them with the TCGA labeled data to train *meth-SemiCancer*. Because it was a single-omic DNAm model, we compared it with our DNAm ones, including RF, SVM, and eSVM. Moreover, to show the effect of the unlabeled samples on *meth-SemiCacner*, we generated its reduced version, *meth-Cancer*, i.e., the neural network only trained on the labeled TCGA data without the unlabeled ones. Corresponding to *meth-SemiCancer*'s ability to utilize unlabeled DNAm data, we also included the results of the multi-omics eSVM here to show eSVM's ability to utilize multi-omics data.

The 5 by 5 cross-validation showed that among the DNAm models, our SVM and eSVM ones performed much better than *meth-SemiCancer* (Fig. S6). SVM-LR and SVM-FLR achieved an error rate of 0.167, and eSVM-LR had one of 0.168. However, *meth-SemiCancer*'s best error rate was 0.172, weaker than the formers. We indeed saw the improvement of *meth-SemiCancer* from its reduced *meth-Cancer* version, whose best error was 0.179, indicating the advantage of unlabeled DNAm data incorporation. However, multi-omics data integration was more effective

because our multi-omics eSVM model achieved an error rate of 0.107. Hence, our package's accuracy and multi-omics incorporation advantage was demonstrated here.

The *methylClass* package can also be applied to DNAm sequencing data

We noted another study using the traditional SVM model to classify pan-cancer data, but the DNAm betas values of this dataset were from the RRBS platform rather than the Illumina DNAm array [17]. Since our package also contained the SVM model, we checked the performance of our SVM and other models on the same RRBS dataset, which had 498 samples and 18 cancer types. The 5 by 5 nested CV showed that SVM was still the best classification method. When used on the *limma*-selected CpG sites in the RRBS data and followed by the LR calibration, it achieved an error rate of 0.167 (Fig. S9). For eSVM and RF, their performance was weaker. The best error rate of eSVM was 0.177 when combined with the *limma* features and the LR or FLR calibration. For RF, its best error was 0.227. In the original RRBS study, the SVM achieved an error rate of 0.19. Hence, this RRBS task was difficult, and all the methods had an error > 0.1.

Supplementary Methods

Data collection and preprocessing

The Infinium 450K BeadChip data on the 2801 CNS tumor samples (DKFZ data) were obtained from the GEO dataset GSE109381 [18]. Then, *minfi* was used to extract beta values and implement control normalization and background correction equal to Genome Studio [19].

After reading the idat files, *minfi*'s function *getControlAddress* was used to find the addresses of 2 control probe sets. One was for the green channel, and the parameter *controlType* of *getControlAddress* was set as *c("NORM_G", "NORM_C")*; the other was for the red channel, and that parameter was set as *c("NORM_A", "NORM_T")*. Then, the means of the green and red control probes were calculated respectively and used to divide the same pre-defined reference value of 10000. Hence, 2 scale factors could be generated to multiply the green and red probe values separately. After that, the 2 colors would have the same scale. Then, the function *bgcorrect.illumina* was used to perform background correction across all the probes.

Next, probes with missing values were imputed. Also, filtering was used to remove the ones covering sex chromosomes or single-nucleotide polymorphism (SNP) sites and those not mapping uniquely to the hg19 human genome or not included on the Illumina EPIC platform. These were implemented with the functions *champ.impute* and *champ.filter* in the *ChAMP* package, with default parameter values [20]. Finally, 428799 qualified probes were obtained.

In addition to these 2801 samples with true labels, GSE109381 contained 1104 samples with only predicted labels from the RF classifier developed by DKFZ. They were preprocessed the same way and used as the validation samples for the classifiers trained in this study. However, because their DKFZ predicted labels inevitably contained noise, to reduce its influence on the validation,

the samples were split into 4 groups according to their DKFZ confidence scores were <= 0.7, > 0.7 & <= 0.8, > 0.8 & <= 0.9, or > 0.9. The group with the highest score had the lowest noise and *vice versus*. In addition, 2 of these 1104 samples were removed because their DKFZ predicted labels were NA. Hence, only 1102 validation samples were used in the analysis.

From TCGA, three 450K DNAm datasets were downloaded with the GDC-client data transfer tool, including the GBM dataset (139 samples), LGG dataset (515 samples), and SARC dataset (260 samples), and their clinical data were also downloaded from TCGA. The missing beta values in the methylation data were imputed using the k-nearest neighbors (KNN) method via the function *impute.knn* in the R package *impute*, with default parameter values.

The DNAm data of the original Sturm study were downloaded from the GEO dataset GSE36278, which contained 136 samples' 450K beta values [14]. Then, their DNAm probes were filtered to remove the Non-CG ones, the ones covering SNP sites, the multiple mapping ones, and the ones located in sex chromosomes.

In detail, the Non-CG probes were the ones with a name not starting from "cg"; the SNP probe list was from the *hm450.manifest.hg19* data frame in the *ChAMPdata* R package, and the ones with a *TRUE* value in its *MASK_general* column was the SNP probes; the multiple mapping probe list was from the *multi.hit* table of the *ChAMPdata* package; the sex chromosome probes were recorded as located in the X and Y chromosomes in the *probe.features* data frame of *ChAMPdata*. All these probes were removed.

After that, the missing beta values in the data were imputed using the function *impute.knn* in the R package *impute*, and the probes with a standard deviation of 0 were removed. Then, the top 8000

most variable ones were selected. They were also contained in the TCGA LGG dataset, so they were used to construct a tSNE embedding space to include all the Sturm and TCGA LGG samples.

The 450K data on the 1077 DKFZ sarcoma samples were downloaded from the GEO dataset GSE140686 [21]. The function *preprocessFunnorm* in *minfi* was used to perform functional normalization directly, with default parameter settings. Next, missing value imputation and probe filtering were implemented similarly to the DKFZ CNS dataset, using the functions *champ.impute* and *champ.filter* in *ChAMP*. Finally, 384629 probes were preserved.

The 428 DKFZ sarcoma samples in the validation dataset were also downloaded from GSE140686 and preprocessed similarly. Also, because their labels were the predictions from the DKFZ sarcoma classifier rather than the true ones, to avoid the influence of the wrong labels from the DFKZ classifier, the samples were stratified according to the DKFZ sarcoma classifier scores.

The 1064 subtype labeled BRCA samples were from TCGA, and their BRCA PAM50 subtype information was from the R package *TCGAbiolinks* [22]. Its command *TCGAquery_subtype("BRCA")* gave a data frame with a column named "BRCA_Subtype_PAM50" and the PAM50 subtype information was extracted there. Then, the samples' 450K/27K DNAm beta data, RNA-seq read counts data, and miRNA-seq read counts data were downloaded. For the DNAm beta data, the function *impute.knn* in *impute* was used to handle the missing values, still with default parameter values. The RNA-seq and miRNA-seq read counts were converted to TPM values.

The unlabeled BRCA samples to train the *meth-SemiCancer* model were from 9 GEO datasets, as indicated in its original study [16]. They were GSE72251, GSE20712, GSE72245, GSE75067, GSE69914, GSE156968, GSE66695, GSE58999, and GSE141441, and their DNAm beta values

were downloaded. If a dataset contained any missing values, *impute.knn* would be used to perform imputation, and if it contained any DNAm probes with a standard deviation of 0, those probes would be removed. Only the shared probes of these datasets were reserved to transfer to *meth-SemiCacner*. Hence, the DNAm samples from the TCGA BRCA dataset were used as the labeled samples, and the GEO samples were used as the unlabeled ones so that the semi-supervised model *meth-SemiCancer* could be constructed. To compare it with other DNAm classifiers, *meth-SemiCancer* was trained and tested in a 5 by 5 nested CV structure, the same as other classifiers.

For the pan-cancer classifier construction, originally, 1712 samples were obtained from various sources. They were downloaded from 10 ArrayExpress datasets and 24 GEO datasets, including E-MTAB-5738, E-MTAB-6450, E-MTAB-6708, E-MTAB-7762, E-MTAB-7854, E-MTAB-8505, E-MTAB-8542, E-MTAB-8660, E-MTAB-8864, E-MTAB-9875, and GSE100850, GSE107946, GSE108982, GSE114989, GSE116699, GSE121377, GSE123601, GSE124413, GSE131350, GSE133395, GSE133556, GSE134089, GSE135017, GSE140686, GSE141039, GSE141363, GSE146003, GSE147667, GSE149282, GSE151067, GSE156299, GSE156358, GSE161692, GSE162554. The whole 1712 samples covered 37 cancer types.

These data were from the EPIC platform and were preprocessed using the *minfi* workflow applied to the DKFZ CNS data above. Still, the function *getControlAddress* was used to get control probes to calculate scale factors, making the green and red probes have the same scale. Then, the function *bgcorrect.illumina* performed background correction. The functions *champ.impute*, and *champ.filter* in *ChAMP* were used to perform missing value imputation and probe filtering. Finally, 746964 quantified probes were preserved.

The RRBS data of 498 pan-cancer samples were downloaded from the GEO dataset GSE230193 [17]. It provided a BedGraph file for each sample, and the beta values of the RRBS CpG sites were

recorded there. The function *read_bed_graph* in the R package *plyranges* was used to read the files, and the function *filter_by_overlaps* was used to find the shared RRBS CpG sites of all the samples. The sites with a standard deviation of 0 would be removed.

Feature selection

The function *mainfeature* in the package was used to select features from the DNAm data for model training, and the top 10000 most variable methylation probes were selected by setting its parameter *subset.CpGs* as 10000. In addition, if it were set as "limma", for each sample class, the significantly differential features between its samples and all the others would be called via *limma* [23], and finally, that of all the classes would be merged, and the top 10000 such features with the smallest adjusted p-values would be kept for classifier construction. In addition, the parameter *subset.CpGs* could also be set as "SCMER", then the *SCMER* algorithm would be called to select markers able to preserve the manifold of original data via $l1$ and $l2$ regularization as, $\min_{\boldsymbol{w}} C + \lambda_1 \|\boldsymbol{w}\|_1 + \lambda_2 \|\boldsymbol{w}\|_2$, where $C = KL(P|Q) = \sum_i \sum_j p_{ij} log \frac{p_{ij}}{q_{ij}}$ was the KL divergence of the sample-sample similarity matrices before (matrix P) and after (matrix Q) feature selection [24]. The regularization constants λ_1 and λ_2 could be set via the function parameters *lasso* and *ridge*, and a combination of *lasso* = 3.25e-7 and *ridge* = 0 was used to select about 10000 *SCMER* probes from 50000 prescreened top variable probes in the DKFZ CNS data. In contrast, they were set as *lasso* = 5e-7 and *ridge* = 0 to select about 10000 *SCMER* probes from 50000 top variable ones from the DKFZ sarcoma data. For the pan-cancer DNAm probe classifier, they were set as *lasso* = *ridge* = 3.5e-7 so that around 10000 DNAm probes were selected as the *SCMER* features from 1198 prescreened pan-cancer samples (originally, the pan-cancer sample number was 1712). For the pan-cancer DNAm pseudo-multi-omics classifier, the original DNAm probes were first split

into 3 groups: gene promoter probes, gene body probes, and other probes. For the promoter probe data, *lasso* = *ridge* = 2.6e-7; for the gene body probe data, *lasso* = *ridge* = 2.3e-7; for the other probe data, *lasso* = *ridge* = 2.3e-7. Finally, around 10000 features were selected for each group. These *lasso* and *ridge* values were determined by grid searches to select about 10000 features from the data.

For the tSNE embedding on the Sturm system DNAm samples, the top 8000 most variable DNAm probes were selected and used for the embedding because Sturm's original study used this number of top variable DNAm probes to define its neural tumor subtypes [14]. In this case, the *subset.CpGs* parameter of *mainfeature* was set as 8000 to get the top 8000 probes. These probes were also contained in the TCGA LGG dataset, so they were extracted from it, and the feature-selected Sturm and LGG samples were embedded in the same tSNE space.

For multi-omics classifier training, two methods in our package were used. One was the eSVM model originally provided by our package. The other was the *MOGONET* model reported previously, a graph convolutional network (GCN) specially developed for multi-omics data [25]. To check their performance on the BRCA multi-omics data, we first conducted feature selection following the method in the original *MOGONET* study and then shared the features with both eSVM and *MOGONET*.

Briefly, for the BRCA DNAm data, only probes contained in the Infinium 27K platform were retained, and the ones with a variance < 0.001 or mean = 0 across the samples were filtered out. For the BRCA mRNA-seq data, the genes with a variance < 0.1 or mean = 0 were removed. For the miRNA-seq data, the miRNA genes with a variance = 0 or mean = 0 were removed. After that, ANOVA was used on each feature across different BRCA subtypes, and only the ones with an adjusted p-value < 0.05 were kept for *MOGONET* and eSVM model construction. In addition, the

PC1 (first principal component) of these filtered data should explain < 50% of the data variance. Finally, because *MOGONET* required that the features in each omic should be within [0, 1], a scaling step was conducted for *MOGONET*, whereas for eSVM, this was skipped.

For the comparison between *meth-SemiCancer* and other DNAm classifiers, the DNAm part of this feature-selected BRCA dataset was used directly. So, the DNAm features were still the ones selected above.

For the package testing on the RRBS dataset, the top 50000 most variable RRBS CpG sites were selected first. Then, *mainfeature* was used on these sites to further select the top 10000, *limma*, and *SCMER* probes via setting its parameter *subset.CpGs* as 10000, "limma", and "SCMER", respectively. The SCMER method's *lasso* and *ridge* parameters were set as *lasso* = *ridge* = 1.28e-6. Then, *maincv* was used on these RRBS beta value data to train and test various classifiers in a 5 by 5 cross-validation manner.

Cross-validation assignment

The functions *makecv* and *cvdata* generated the 5 by 5 nested CV structure. First, the whole samples were randomly divided into 5 sets. For each outer CV loop, one of these 5 sets was used as the testing, whereas the other 4 were merged as the training. Then, in each training set, the samples were further divided into 5 subsets to generate 5 inner CV loops. Finally, 5 outer loops were made, each derived 5 inner ones. The outer loops evaluated the model's generalization, whereas the inner loops helped avoid over-fitting during calibration.

The functions *makecv* and *cvdata* also provided a parameter named *normalcv*, and if setting it as TRUE, only the outer loops would be generated, which formed a normal CV structure.

Machine learning model training

The function *maincv* was used to train the machine learning models in a CV framework. It used the CV sample assignment result from the function *makecv* to divide the methylation matrix into several subsets, and for the training set of each CV loop, feature selection and classifier construction were performed.

The feature selection step was completed by calling the function *mainfeature*. Then, the top variable, *limma*, or *SCMER* features, were selected.

Next, with the features, a machine learning model was trained to classify the samples into their true labels provided. The model type could be chosen via the parameter *method* and from the candidates of "RF", "XGB", "ENet", "SVM", "eSVM", "eNerual", and "MOGONET". The former 4 were based on the R packages *randomForest*, *xgboost*, *glmnet*, and *e1071,* respectively. Because of the sparsity of the DNAm samples in the super-high dimension space, the "SVM" method only used the linear kernel, which had been proved to be more accurate than other kernels in this case [26]. On the other hand, for the "eSVM", "eNerual", and "MOGONET" methods, they were different from the formers because of their capability of handling not only single-omic but also multi-omics data due to the bagging framework they adopted.

For RF, it was constructed following the DKFZ RF classifier published previously and included 2 runs of RF [18], both of which used 500 decision trees as base learners. The first one randomly sampled $\sqrt{p}$ features for each tree from the total p features in the whole dataset and then ranked them according to their importance measured by the decrease in model accuracy. Then, the second RF run fine-tuned the results via training the model using the top 200 most important features.

For eSVM, if it accepted a single-omic dataset, such as a DNAm dataset, it would randomly split its features into 10 groups. Hence, each group included around 1/10 of the original features. These

10 feature groups were then used to train 10 base learners in the bagging framework, and for each base learner, bootstrapping was performed on all the samples to generate a sample set with the same size but with bootstrapped replicates. Hence, each base leaner contained the same sample number as the original data, but its feature number was reduced to around 1/10 of the original one. Then, an SVM was trained for each base learner, and the final 10 SVM base learners would be ensembled into an eSVM model.

For linear kernel SVM, its model could be expressed as $f(\boldsymbol{x}) = \boldsymbol{w}^{\boldsymbol{T}}\boldsymbol{x} + b$, where $\boldsymbol{x}$ was a sample vector with d elements, corresponding to a sample with d features, such as d methylation probes, so that each element in the vector represented the value of one probe. After calculation with $\boldsymbol{w}$ and b in the formula, the final value $f(\boldsymbol{x})$ indicated the predicted class of sample $\boldsymbol{x}$, and the key here was to learn $\boldsymbol{w}$ and b from the data. Actually, they could be calculated following the formulas: $\boldsymbol{w} = \sum_{i=1}^{m} \alpha_i y_i \boldsymbol{x_i}$ and $b = \frac{1}{m}\sum_{j=1}^{m}(\frac{1}{y_j} - \sum_{i=1}^{m} \alpha_i y_i \boldsymbol{x_i^T} \boldsymbol{x_j})$, where $\boldsymbol{x_i}$ and $\boldsymbol{x_j}$ were the vectors for the i-th and j-th samples in the dataset. The whole dataset contained m samples, and each sample had d features, so the indices i and j ranged from 1 to m, and the vectors $\boldsymbol{x_i}$ and $\boldsymbol{x_j}$ contained d elements. On the other hand, y_i and y_j were the scalars representing the true labels of $\boldsymbol{x_i}$ and $\boldsymbol{x_j}$. It should be noted that in the formulas, $\boldsymbol{x_i}$, $\boldsymbol{x_j}$, y_i, and y_j were known from the dataset, and the only unknown scalar was α_i, which was the Language multiplier for the i-th sample when deriving the formulas. Hence, to calculate the values of $\boldsymbol{w}$ and b, α_i must be calculated. Then, $\boldsymbol{w}$ and b could be obtained via the formulas, and the model $f(\boldsymbol{x}) = \boldsymbol{w}^{\boldsymbol{T}}\boldsymbol{x} + b$ would be determined. For α_i, it could be solved from the dual problem of SVM, which was, $\max_{\boldsymbol{\alpha}} \sum_{i=1}^{m} \alpha_i - \frac{1}{2}\sum_{i=1}^{m}\sum_{j=1}^{m} \alpha_i \alpha_j y_i y_j \boldsymbol{x_i^T} \boldsymbol{x_j}\ s.t. \sum_{i=1}^{m} \alpha_i y_i = 0, 0 \le \alpha_i \le C, i = 1, 2, \ldots, m$, where C was the regularization constant to control the deviation of the model when training it, and would be defined

by the user. Hence, in this dual problem, the values of $\boldsymbol{x_i}$, $\boldsymbol{x_j}$, y_i, y_j and C were known, and the values of α_i and α_j were unknown and needed to be solved from it. This was achieved by the SMO (sequential minimal optimization) algorithm, which simplified it to a quadratic programming problem with analytic expression. However, when solving the quadratic programming, the $\boldsymbol{x_i^T x_j}$ part in the formula above still needed to be calculated, and after that, α_i and α_j values could be obtained, and then the $\boldsymbol{w}$ and b parameters, as well as the final SVM model could be determined.

To calculate the inner product $\boldsymbol{x_i^T x_j}$, because both vectors contained d elements, totally d times of multiplication and $d - 1$ times of summation would be needed. In the case of a DNAm dataset with the top 10000 most variable DNAm probes, the d value was 10000, and so when training a traditional SVM model from it, 10000 times of multiplication and 9999 times of summation would be conducted. However, for an eSVM model, because the feature sampling process randomly split the 10000 probes into 10 base learners, with each including around 1000 probes, when constructing an SVM base learner, only 1000 times of multiplication and 999 times of summation would be used, largely reducing the computational time. Hence, eSVM used the feature sampling step of its bagging framework to relieve the time-consuming problem of SVM, and with a parallelization backend, the running time of the whole ensemble would be similar to a single base learner.

On the other hand, because of the feature reduction, an SVM base learner's accuracy would be weaker than a traditional SVM trained from all the features. However, this could be compensated by the aggregation step of the whole ensemble. Although the random sampling decreased the feature number of each base learner, it increased the divergence between base learners because different base learners would have largely different features sampled from the original set. In this case, the error rate of the final ensemble model could be calculated theoretically as $P(F(\boldsymbol{x}) \neq y) =$

$\sum_{k=0}^{[\frac{N}{2}]}\binom{N}{k}(1-error)^k\, error^{N-k}$, where y was the true label of sample $\boldsymbol{x}$, and $F(\boldsymbol{x})$ was the predicted label from the ensemble model, so $P(F(\boldsymbol{x}) \neq y)$ was the probability that the ensemble's prediction was different from the true label, i.e., its error rate. Then, for the right-hand side of the formula, N was the base learner number in the whole ensemble model, k was the number of base learners giving a correct prediction, and $error$ was the averaged error rate of all the base learners, so this part indicated the probability that less than half of the base learners gave a correct prediction, which was also the error rate of the whole ensemble, $P(F(\boldsymbol{x}) \neq y)$. Then, the upper limit of this probability could be derived based on Hoeffding's inequality as $P(F(\boldsymbol{x}) \neq y) = \sum_{k=0}^{[\frac{N}{2}]}\binom{N}{k}(1-error)^k\, error^{N-k} \leq \exp(-\frac{1}{2}N(1-2*error)^2)$, and it could be seen here that as the base learner number N increased, the error limit $\exp(-\frac{1}{2}N(1-2*error)^2)$ would decrease exponentially, meaning that increasing base learner would overcome their low accuracy and improve the ensemble's performance. However, this formula actually assumed that the base learners were independent of each other, which was not the case in the real situation, but if the divergence between them were large, the situation would be closer to an independent one. Hence, in eSVM, because its feature sampling step increased the base learner divergence, the aggregation step could be more effective in compensating for the accuracy impairment.

In the real case, this aggregation was fulfilled by base learner weighting, i.e., for each base learner, its misclassification error would be calculated. If one had an error >= 0.5, it would be dropped, whereas for that < 0.5, it would be kept to construct the whole ensemble, and a weight would be assigned to it as $0.5 * \frac{1-error}{error}$. After collecting 10 qualified base learners, their weights would be further scaled to the sum of 1. The raw result of the whole ensemble was the weighted sum of these base learners. The weighting process could be expressed as $F(\boldsymbol{x}) = \sum_{k=1}^{N}\beta_k * f_k(\boldsymbol{x})\ s.t.\ \beta_k >$

$0, \sum_{k=1}^{N} \beta_k = 1, P(f_k(\boldsymbol{x}) \neq y) < 0.5$, where $f_k(\boldsymbol{x})$ was the prediction result from the k-th base learner, and β_k was the weight assigned to it. The ensemble contained totally N base learners, and its result $F(\boldsymbol{x})$ was the weighted sum of different $f_k(\boldsymbol{x})$.

Hence, in eSVM, the feature sampling step accelerated the computational speed. For the side effect of accuracy reduction, the sampling step made compensation when combined with the weighting strategy.

After weighting, the model's accuracy was further enhanced via calibration, which trained a new model to predict the true labels from the current model's prediction. In our package, the current prediction was generated by the function *maincv*, and another function, *maincalibration*, achieved the calibration, providing 3 methods to train the calibration model, including LR, FLR, and MR. This process was after the weighting step of *maincv*, and the whole model could be expressed as $H(\boldsymbol{x}) = h(F(\boldsymbol{x})) = h\left(\sum_{k=1}^{N} \beta_k * f_k(\boldsymbol{x})\right) s.t. \beta_k > 0, \sum_{k=1}^{N} \beta_k = 1, P(f_k(\boldsymbol{x}) \neq y) < 0.5$, where h represented the calibration model, and its input was $F(\boldsymbol{x})$, which was the output of the weighting step in *maincv*. On the other hand, its output $H(\boldsymbol{x})$ was the final prediction. The calibration model h functioned to fine-tune the raw result $F(\boldsymbol{x})$, and then the final result $H(\boldsymbol{x})$ was obtained.

Hence, weighting and calibration ensured the model's accuracy after acceleration of computational speed. In addition, eSVM also had another advantage: the expanded application to multi-omics data, still benefiting from the ensemble framework. For multi-omics, the feature sampling and ensemble steps on single-omic could also be used, and eSVM also generated 10 base learners via feature sampling. However, the difference was that in the single-omic situation, all the base learners were from the same omic, just with different feature sets, but in the multi-omics case, different base learners might be from different omics.

In detail, *maincv* accepted the multi-omics data as a single matrix with rows representing samples and columns representing features from various omics, and another parameter, *multiomicsnames,* was used to indicate which features were from which omics. It accepted a vector whose length was the same as the matrix's column number, i.e., the feature number in the whole dataset. Each element in the vector was the omic name of its corresponding feature in the matrix, such as "methylation", "RNA", "miRNA", etc. Then, within *maincv*, the matrix's columns were separated, and the ones with the same omic name were combined, so finally, several small matrices would form representing different single-omic data.

Then, each small matrix went through the feature sampling step of *maincv*, with several feature groups generated, and all of them totally generated 10 groups. Hence, for each group, its features were from the same small matrix, i.e., single-omic data, but for different groups, their features might be from different ones. The number of groups generated from each omic was determined by the feature number ratio between different omics, so an omic with more features would have its features divided into more groups, and one with fewer features would only be divided into a few groups. In this manner, the feature number of each group would be almost the same, and a total of 10 feature groups would form. This total number of 10 could be changed via the parameter *learnernum*, so > 10 feature groups, i.e., base learners, could also be generated.

After this feature step, the next sample bootstrapping step was the same as the single-omic case, so the multi-omics data were finally split into different base learner datasets, and all of them had the same sample number and similar feature numbers. However, for the features, although the ones within the same base learner were from the same omic, the ones in different base learners might be from different omics. Hence, the inter-base-learner divergence had 2 sources. One was the feature sampling step, which made base learners from the same omic have a large feature

difference, and this was also the only divergence source when using eSVM on single-omic data. However, for multi-omics, if 2 base learners were from different omics, their omics difference was also a divergence source, which was more important than sampling.

Computationally, this larger divergence from multi-omics made the base learners more independent of each other, and for the former error limit $\exp(-\frac{1}{2}N(1-2*error)^2)$, its independence assumption could be fulfilled better. Hence, the ensemble's aggregation step could improve the model's accuracy more effectively.

Biologically, multi-omics data provided a more holistic view of cancer and its diagnosis because oncogenesis involved complex rearrangements at the genetic, epigenetic, transcriptional, and proteomic levels. In contrast, single-omic data only captured part of this complex regulatory network. Hence, integrating multi-omics could improve the accuracy of cancer classification, and eSVM's ensemble framework enabled it.

Then, the ensemble results would be transferred to *maincalibration* to further enhance the accuracy via calibration, which was the same as the single-omic case.

In addition to eSVM, the eNeural model was constructed using a similar ensemble framework, and the only difference was that eNeural used MLP neural networks to train the base learners. Each base learner contained 2 hidden layers with a hidden node number as 1/10 and 1/20 of its input features. For other hyperparameters of MLP, the activation function was ReLU, the epoch number was 10, the initial momentum was 0, the learning rate decay factor was 0.99, the cycle fraction for scoring was 0.025, and the metric for early stopping was misclassification error, with a relative tolerance of 0.01. However, if the function parameter *gridsearch* were set as TRUE, the hidden

layer depth and size, activation function, epoch number, initial momentum, and learning rate decay factor would be selected via grid search.

Another multi-omics model that could be trained via *maincv* was *MOGONET* [25]. It treated each omic as one base learner and applied a GCN neural network to train it. After that, a fully connected neural network was used to integrate all the base learner results and their interaction terms to predict the sample labels. However, in *maincv*, 2 modifications were made to this original *MOGONET*. The first was that its base learner number was made more flexible. For each omic, more than one GCN model could be trained this time by dividing it into different feature groups, as in eSVM and eNeural, so single-omic data could also be treated as multi-omics by *MOGONET*. The second modification was on its omics integration step. In the original model, this step calculated the interaction terms among the classification results of different omics. However, such terms would increase sharply as the sample class number increased, making it impossible to apply to tasks with a large class number. In the case of a 3-omics dataset with 5 sample classes, each omic base learner calculated the posterior probability of a sample belonging to different classes, i.e., for each sample, a vector with 5 elements would be generated. Each element represented its probability of belonging to each of the 5 classes. In the original *MOGONET* model, the 3 omics generated 3 base learners and also 3 of such vectors, and all of them would be transferred to the integration step. Then, the model found all the class probability combinations between different omics and used them as interaction terms. This combination number was $5^3 = 125$, so it generated 125 interaction terms. Finally, these 125 terms were used to input the fully connected neural network to predict the true labels. This process was acceptable for tasks with small omics and sample class numbers, such as the case here. However, for many cancer classification tasks, the class number was very large, leading to a huge interaction term number. And even for a task

covering 10 cancer classes and 3 omics, the interaction term number was $10^3 = 1000$. Hence, these exponentially increased interaction terms would largely inhibit computational efficiency. To solve this problem, in our *maincv* function, a restriction was added to the integration step. If the class number were > 2, the integration step would only use the original GCN results without calculating their interaction terms. All the model parameters used for *MOGONET* were the same as its original study, such as a pretraining epoch number of 500 and a training epoch number of 2500, and each type of omics data was scaled to the range of [0, 1] before training.

Because *maincv* trained the models on a CV framework, it aimed to evaluate the model performance via the predictions on testing sets. However, to train a model that could be used on validation or external datasets, another function, *maintrain,* should be used. It performed machine learning similar to *maincv* but on the whole dataset. In addition, the downstream calibration steps were also included in *maintrain*, so models with different calibration methods could be returned.

Model calibration and performance evaluation

The function *maincalibration* was used to calibrate the raw results from *maincv* and improve the accuracy. It provided 3 methods to complete this via re-predicting the true labels using the raw model predictions. The first method was LR, and the second was FLR fulfilled via the R package *brglm*. The third method was MR based on the package *glmnet*. In addition, *maincalibration* also evaluated the performance of the raw and calibrated models with 3 metrics, including misclassification error, Brier score, and cross-entropy loss (mLogloss). The whole process was as follows.

First, *maincalibration* inherited the CV structure used by *maincv* during the previous raw model training. In the case of a normal 5-fold CV structure, *maincalibration* constructed one calibration

model from the training set of each CV loop. The response variable was the true labels of the training samples, and the predictor variable was their posterior probabilities of belonging to different classes, which was predicted by the raw models trained by *maincv*. It should be noted that these posterior probabilities were predicted not just from 1 model but from 4 models. It was because, during the 5-fold CV, *maincv* generated 5 raw models, each trained from one CV loop. Then, for the current calibration CV loop, its training set was equal to the combination of the other 4 CV loops' testing sets. Correspondingly, the posterior probabilities of this training set were obtained by combining all the testing set probabilities from those 4 CV loops, which meant each of them provided the probabilities of a part of the training samples, and all of them could cover the whole. After getting these probabilities, they were used as the predictor variable, and the true labels were used as the response variable. When transferred to the 3 calibration methods, 3 calibration models were generated from the current training set. Each was then used on the CV loop's testing samples to predict their calibrated classifications. Then, this result was compared with the true labels, and the misclassification error, Brier score, and mLogloss could be calculated to evaluate the model performance.

On the other hand, if the CV structure was not a normal 5-fold CV structure as here, but a nested one, these steps were almost the same, and the only difference was that the training samples' posterior probabilities were not combined from other outer CV loops, but from the inner CV loops, which could reduce the over-fitting risk.

For the evaluation metrics, Brier score and mLogloss were calculated as, $Brier = \frac{1}{n}\sum_{i=1}^{n}\sum_{j=1}^{m}(p_{i,j} - y_{i,j})^2$ and $mLogloss = -\frac{1}{n}\sum_{i=1}^{n}\sum_{j=1}^{m} y_{i,j}\log(p_{i,j})$, where $p_{i,j}$ was the model predicted probability that sample i belonged to class j, and $y_{i,j}$ was the true label, and if sample i belonged to class j, its value was 1; otherwise, it was 0.

Brier score was an evaluation metric checking the goodness of prediction probabilities. It was similar to the mean squared error but only applied to probabilities. On the other hand, mLogloss, also known as cross-entropy, calculated the entropy between the prediction probabilities and the true labels. Unlike these 2 metrics, error rate did not involve any probability. It directly calculated the percentage of a model's misclassification. Hence, error rate was the simplest metric but was also the most direct measurement of model performance.

Internal validation indices calculation

For the internal validation indices (Silhouette, Calinski-Harabasz, and Davis-Bouldin), they were calculated as, $Silhouette = \frac{1}{k}\sum_{i=1}^{k}\left\{\frac{1}{|C_i|}\sum_{x \in C_i}\frac{b(x)-a(x)}{\max[b(x),a(x)]}\right\}$, where $a(x) = \frac{1}{|C_i|-1}\sum_{y \in C_i, x \neq y} d(x,y)$, $b(x) = min_{j, j \neq i}\left[\frac{1}{|C_i|}\sum_{y \in C_i} d(x,y)\right]$. $Calinski - Harabasz = \frac{\sum_i^k |C_i| d^2(c_i,c)/(n-1)}{\sum_i^k \sum_{x \in C_i} d^2(x,c_i)/(n-k)}$ and $Davis - Bouldin = \frac{1}{k}\sum_i^k max_{j, j \neq i}\left\{\left[\frac{1}{|C_i|}\sum_{x \in C_i} d(x,c_i) + \frac{1}{|C_j|}\sum_{x \in C_j} d(x,c_j)\right] / d(c_i,c_j)\right\}$, where C_i was the i-th cluster (here was class), c_i was the center of C_i, c was the center of the whole data, and $d(x,y)$ was the distance between x and y.

Prediction of new samples

The function *mainpredict* accepted the data matrix of new samples and used the raw and calibrated models to predict the sample labels.

Because, in some cases, new samples may be from classes not involved in model training, *mainpredict* specially included a parameter named *unseenlabelcutoff*. It would serve as a probability cutoff if it were set as a float number between 0 and 1. Hence, if a sample's predicted probability of belonging to all the observed classes were less than this cutoff, such as 0.5, its label

would be predicted as "unknown". On the other hand, if *unseenlabelcutoff* were set as *NULL*, which was its default value, all the new samples would be deemed from classes observed during training, and their labels would be the ones with the largest predicted probabilities without any cutoff considered. However, in both cases, the function would also return the samples' probability matrix so the users could check them directly.

For the case studies of DKFZ CNS sample validation, DKFZ sarcoma validation, and TCGA LGG, GBM, and SARC datasets, the new sample labels were predicted by the 6 selected SVM/eSVM-MR models with *mainpredict*, followed by an ensemble step to aggregate the 6 models' results. The ensemble weight of each model was calculated from its misclassification error on the 5 by 5 CV as $0.5 * \frac{1-error}{error}$. Then, the 6 models' weights would be further scaled to the sum of 1. After that, the models' sample label distribution matrices were weighted accordingly and merged to get a final one. For each sample, its label was assigned as the one with the largest probability value in this matrix, and for the TCGA LGG, GBM, and SARC datasets, only the labels with a predicted sample number > 10 were preserved. In contrast, others were combined as an "others" class.

DNA methylation probe distribution analysis

The function *multipledistribution* generated the genomic distribution results of the selected methylation probes. It attributed the probes to different genomic regions according to their annotation information of "Relation_to_Island" and "UCSC_RefGene_Name" in the 450K or EPIC data and checked the enrichment of each kind of probe in the selected set using Fisher's exact test.

Gene functional enrichment analysis

The selected probes with a TSS200, TSS1500, and 1stExon localization were mapped to their corresponding genes, with the function *togene* in the package. It calculated a gene's methylation beta value by averaging the probe values located in its specific regions, defined by the function's parameter *group450k850k*. In this study, it was set as *c("TSS200", "TSS1500", "1stExon")*, so the probes in these regions were used to calculate the gene beta values. The probe beta values were subsetted from the original DNAm data across different samples and then transferred to the function. In the gene functional enrichment case, the final gene beta values were not needed, but the gene names the probes mapped to were required, and they were the row names of the gene beta value matrix returned by *togene*.

Then, the package *enrichR* was used to conduct functional enrichment analysis [27]. Its function *enrichr* accepted the gene names via its parameter *genes*, and the other parameter *databases* was used to specify the gene functional databases for the enrichment analysis. In this study, it was set as *c("GO_Biological_Process_2018", "GO_Molecular_Function_2018", "BioPlanet_2019", "WikiPathways_2019_Human", "KEGG_2019_Human", "BioCarta_2016", "Reactome_2016", "NCI-Nature_2016", "Panther_2016")*, so *enrichr* found the genes' enriched functions from these databases. Its returned result gave several parameters, including *Adjusted.P.value* and *P.value*, and if a functional term had an *Adjusted.P.value* < 0.05, its enrichment was deemed significant. However, if < 10 terms in the results reached this criterion, *P.value* < 0.01 would be used as a relatively loose criterion to determine the significant functional terms.

Multi-omics data visualization

The *mainjvisR* function in the package performed the joint tSNE embedding on the multi-omics data. Also, it showed the sample positions in this embedding so that users could visualize them directly.

It followed the *JVis* method to minimize $C = \sum_{i=1}^{k} \alpha_i KL(P_i|Q) + \lambda \sum_{i=1}^{k} \alpha_i log\alpha_i$, where P_i was the sample-sample similarity matrix of the i-th modality, calculated from its original high-dimensional data, and Q was the sample-sample similarity matrix of the joint embedding, calculated from the reduced low-dimensional data. Then, $KL(P_i|Q)$ was the KL divergence between them. KL divergence was originally a statistical distance measuring the difference between 2 distributions, and its application to the sample-sample similarity matrices here indicated any inter-sample position change after mapping them from the high dimensional space to the low one. Another parameter α_i was the weight for the i-th modality subject to the constraint $\alpha_i \geq 0$ and $\sum_{i}^{k} \alpha_i = 1$, so the first part of the formula, $\sum_{i=1}^{k} \alpha_i KL(P_i|Q)$, was the weighted sum of the KL divergences, i.e., inter-sample position differences, between each high-dimensional modality P_i and the low-dimensional joint embedding Q, and *JVis* aimed to find the optimal Q to minimize this difference after the dimension reduction. The second part, $\lambda \sum_{i=1}^{k} \alpha_i log\alpha_i$, in the formula was a regularization part, and its coefficient λ was the regularization parameter functioning to prevent the joint embedding from being biased towards individual modalities and needed to be set by the users [28].

Similar to this method for joint tSNE, joint UMAP could also be generated via *mainjvisR*, and the only difference was that the KL divergence in the formula was changed to cross-entropy. When the number of modalities k was 1, i.e., only 1 omic was used for the embedding, the joint results were exactly the normal tSNE and UMAP results.

When using this method on the BRCA multi-omics data, the RNA, methylation, and miRNA data were included in an R list; each was a list element. Then, it was transferred to *mainjvisR* via the parameter *datlist*. In addition, the sample labels were transferred via the parameter *labels*, and all other parameters were set as their default values. Finally, the function returned the scatter plots showing the samples' coordinates and positions in the joint tSNE and joint UMAP embeddings. Hence, it fulfilled the multi-omics data visualization function. If users wanted to generate plots with their plotting preference, *mainjvisR* also returned the tables recording the samples' coordinates values in the embeddings and could be used to plot directly.

Pan-cancer data filtering

Several steps were used to remove the noisy samples from the original 1712 pan-cancer samples. First, the function *mainfeature* was used on the probe methylation data (746964 probes across 1712 samples) to select the top 10000 most variable features, the *limma* features, and the *SCMER* features. Then, these 3 kinds of probes were combined to get their union, and the DNAm data of these union probes were transferred to the function *mainjvisR* to get a tSNE embedding.

In detail, *mainfeature* accepted the beta value matrix of the whole dataset and its parameter *subset.CpGs* was set as 10000, "limma", or "SCMER" to select the corresponding feature sets. In the *SCMER* case, another 2 parameters, *lasso* and *ridge*, needed to be set specially, which controlled the $l1$ and $l2$ regularization constants for the *SCMER* algorithm. For this pan-cancer dataset, both of them were set as 3.5e-7. Then, all other parameters were left as their default values. After obtaining these 3 feature groups, their union was used as the final feature set, and their beta matrix was transferred to the function *mainjvisR*. Its result was the DNAm samples' coordinates in tSNE embedding. All the parameters of *mainjvisR* were left as their default values when running it.

Next, DBSCAN (density-based spatial clustering of applications with noise) was used on this tSNE embedding to cluster and filter the samples. This was performed with the functions *clustergrid* and *labelclusters* in the package. One DBSCAN cluster could contain samples with different cancer-type labels. If > 75% of its samples were from the same cancer type, this cluster would be deemed a dominant cluster, and its label was the label of these dominant samples. However, if all of its sample labels had a proportion <= 75%, this cluster would be deemed noisy and discarded. In contrast, a dominant cluster would be retained, but the minor samples in it with a cancer-type label different from the dominant one would be removed from the cluster. Hence, *clustergrid* filtered out noisy clusters with no dominant labels and noisy samples in the dominant clusters. In addition, the samples not belonging to any DBSCAN clusters would also be removed. Finally, each cluster would contain samples from one cancer type. In some cases, different clusters might have the same labels, and they would be merged.

Because this filtering step depended on the DBSCAN result, to find the optimal parameters for DBSCAN, *clustergrid* would perform a grid search for the epsilon and the minimum cluster point values for DBSCAN. The best combination of these 2 parameters was the one retaining the most samples after the filtering. The best parameters for the 1712 pan-cancer samples were epsilon = 1.21 and minimum cluster point = 3, and they could retain 1198 of the 1712 samples.

Concretely, the tSNE result of *mainjvisR* and the samples' true labels were transferred to *clustergrid* to perform the grid search for DBSCAN. The parameter *epses* of this function accepted the candidate values for epsilon and was set as a number sequence of *seq(1, 5, 0.01)*. Another parameter, *minPtses*, was the candidate values of the minimum cluster points and was also a sequence, *seq(3, 21, 1)*. Hence, different combinations of these *epses* and *minPtses* values would be tested. In addition, the parameter *cutoff* was set as 0.75, meaning the dominant clusters should

have > 75% identically labeled samples. Then, *clustergrid* returned the optimal parameter combination able to keep most samples. Next, the function *labelcluster* accepted these results and outputted the final IDs of the retained samples. Its parameters *tsnedat* received the tSNE result of *mainjvisR*, and *eps*, *minPts*, and *cutoff* took the optimal DBSCAN parameters output by *clustergrid*, which were 1.21, 3, and 0.75, respectively.

These retained samples covered 33 pan-cancer clusters, including Acral lentiginous melanoma (16 samples), Acute myelogenous leukemia (67 samples), Adrenal adenoma (4 samples), Atypical teratoid/rhabdoid tumor (67 samples), B-acute lymphoblastic leukemia (61 samples), Breast adenocarcinoma (27 samples), Chondroblastoma (8 samples), Chondrosarcoma (24 samples), Chordoma (55 samples), Clear cell sarcoma (kidney) (11 samples), Colorectal adenocarcinoma (5 samples), Colorectal adenoma (87 samples), Endometrial stromal sarcoma (23 samples), Ewings sarcoma (17 samples), Gastrointestinal stromal tumor (22 samples), High-grade serous adenocarcinoma (78 samples), Intraductal tubulopapillary neoplasm (24 samples), Langerhans cell histiocytosis (11 samples), Leiomyosarcoma (19 samples), Lung adenocarcinoma (36 samples), Malignant peripheral nerve sheath tumor (18 samples), Malignant rhabdoid tumor (62 samples), Meningioma (86 samples), Myxofibrosarcoma (37 samples), Neuroendocrine tumor (40 samples), Osteosarcoma (70 samples), Papillay carcinoma (10 samples), Paraganglioma/pheochromocytoma (10 samples), Pituitary adenoma (62 samples), Rhabdomyosarcoma (35 samples), Solitary fibrous tumor (12 samples), Squamous cell carcinoma (14 samples), and T-lymphoblastic leukemia/lymphoma (80 samples).

For this clean sample set, before transferring it to the function *maincv* to construct and evaluate the pan-cancer classifier, an up-sampling step was applied to them via the function *balancesampling*. This was because, after the filtering, the sample sizes of some cancer types

became very small, which made the sample sizes among different cancer types more imbalanced. This could be overcome by *balancesampling*. It synthesized some samples for the cancer types with a sample size < 10 so that they finally contained 10 samples, and the imbalance could be relieved. The sample synthesis of *balancesampling* was fulfilled via the SMOTE (Synthetic Minority Over-sampling Technique) method. For a cancer type with < 10 samples, it randomly selected one sample of this type and then searched for its 5 nearest neighbors with the same label, and this search was based on the top 50000 most variable probes of the whole dataset. After identifying the neighbors, *balancesampling* randomly selected one of them and calculated the vector recording the feature value differences between the original sample and the selected neighbor. Then, a random number between 0 and 1 would be generated to multiply this vector, and the new vector would be added to the original sample vector. The result was the synthesized sample, and its label was assigned as identical to the original sample.

The *balancesampling* function accepted the beta value matrix and the true labels via its parameters *dat* and *labels*. In addition, the parameter *cutoff* was set as 10, so the SMOTE up-sampling was used on classes with < 10 samples, and *k* was set as 5 to make SMOTE select the 5 nearest neighbors of a specific sample to perform the synthesis. All other parameters were left as their default values.

Finally, all the samples were transferred to the function *maincv*, and their top10k, *limma*, and *SCMER* features were used to establish the pan-cancer classifiers, respectively.

References

1. Robert SM, Sontheimer H. Glutamate transporters in the biology of malignant gliomas, Cellular and Molecular Life Sciences 2014;71:1839-1854.
2. Lyons SA, Chung WJ, Weaver AK et al. Autocrine Glutamate Signaling Promotes Glioma Cell Invasion, Cancer Res 2007;67:9463-9471.
3. Ye ZC, Sontheimer H. Glioma cells release excitotoxic concentrations of glutamate, Cancer Res 1999;59:4383-4391.
4. Choi J, Stradmann-Bellinghausen B, Yakubov E et al. Glioblastoma cells induce differential glutamatergic gene expressions in human tumor-associated microglia/macrophages and monocyte-derived macrophages, Cancer Biology & Therapy 2015;16:1205-1213.
5. Liu J, Lichtenberg T, Hoadley KA et al. An Integrated TCGA Pan-Cancer Clinical Data Resource to Drive High-Quality Survival Outcome Analytics, Cell 2018;173:400-416.e411.
6. Lang GA, Iwakuma T, Suh Y-A et al. Gain of Function of a p53 Hot Spot Mutation in a Mouse Model of Li-Fraumeni Syndrome, Cell 2004;119:861-872.
7. Kim MP, Zhang Y, Lozano G. Mutant p53: Multiple Mechanisms Define Biologic Activity in Cancer, Frontiers in Oncology 2015;5.
8. Jesionek-Kupnicka D, Szybka M, Malachowska B et al. TP53 promoter methylation in primary glioblastoma: relationship with TP53 mRNA and protein expression and mutation status, DNA Cell Biol 2014;33:217-226.
9. Maßberg D, Simon A, Häussinger D et al. Monoterpene (−)-citronellal affects hepatocarcinoma cell signaling via an olfactory receptor, Archives of Biochemistry and Biophysics 2015;566:100-109.
10. Weber L, Maßberg D, Becker C et al. Olfactory Receptors as Biomarkers in Human Breast Carcinoma Tissues, Frontiers in Oncology 2018;8.
11. Neuhaus EM, Zhang W, Gelis L et al. Activation of an Olfactory Receptor Inhibits Proliferation of Prostate Cancer Cells *, Journal of Biological Chemistry 2009;284:16218-16225.
12. Cho HJ, Koo J. Odorant G protein-coupled receptors as potential therapeutic targets for adult diffuse gliomas: a systematic analysis and review, BMB Reports 2021;54:601-607.
13. Wang Q, He Z, Chen Y. Comprehensive Analysis Reveals a 4-Gene Signature in Predicting Response to Temozolomide in Low-Grade Glioma Patients, Cancer Control 2019;26:1073274819855118.
14. Sturm D, Witt H, Hovestadt V et al. Hotspot mutations in H3F3A and IDH1 define distinct epigenetic and biological subgroups of glioblastoma, Cancer Cell 2012;22:425-437.
15. Álvarez-Torres MdM, López-Cerdán A, Andreu Z et al. Vascular differences between IDH-wildtype glioblastoma and astrocytoma IDH-mutant grade 4 at imaging and transcriptomic levels, NMR in Biomedicine 2023;36:e5004.
16. Choi JM, Park C, Chae H. meth-SemiCancer: a cancer subtype classification framework via semi-supervised learning utilizing DNA methylation profiles, BMC Bioinformatics 2023;24:168.
17. Zhang S, He S, Zhu X et al. DNA methylation profiling to determine the primary sites of metastatic cancers using formalin-fixed paraffin-embedded tissues, Nature Communications 2023;14:5686.
18. Capper D, Jones DTW, Sill M et al. DNA methylation-based classification of central nervous system tumours, Nature 2018;555:469-474.
19. Fortin J-P, Triche TJ, Jr, Hansen KD. Preprocessing, normalization and integration of the Illumina HumanMethylationEPIC array with minfi, Bioinformatics 2016;33:558-560.
20. Tian Y, Morris TJ, Webster AP et al. ChAMP: updated methylation analysis pipeline for Illumina BeadChips, Bioinformatics 2017;33:3982-3984.

21. Koelsche C, Schrimpf D, Stichel D et al. Sarcoma classification by DNA methylation profiling, Nature Communications 2021;12:498.
22. Colaprico A, Silva TC, Olsen C et al. TCGAbiolinks: an R/Bioconductor package for integrative analysis of TCGA data, Nucleic Acids Res 2015;44:e71-e71.
23. Ritchie ME, Phipson B, Wu D et al. limma powers differential expression analyses for RNA-sequencing and microarray studies, Nucleic Acids Res 2015;43:e47-e47.
24. Liang S, Mohanty V, Dou J et al. Single-cell manifold-preserving feature selection for detecting rare cell populations, Nature Computational Science 2021;1:374-384.
25. Wang T, Shao W, Huang Z et al. MOGONET integrates multi-omics data using graph convolutional networks allowing patient classification and biomarker identification, Nature Communications 2021;12:3445.
26. Maros ME, Capper D, Jones DTW et al. Machine learning workflows to estimate class probabilities for precision cancer diagnostics on DNA methylation microarray data, Nature Protocols 2020;15:479-512.
27. Kuleshov MV, Jones MR, Rouillard AD et al. Enrichr: a comprehensive gene set enrichment analysis web server 2016 update, Nucleic Acids Res 2016;44:W90-97.
28. Do VH, Canzar S. A generalization of t-SNE and UMAP to single-cell multimodal omics, Genome Biology 2021;22:130.
