# Supplementary material for: methylClass: an R package to construct DNA methylation-based classification models
Source: Brief Bioinform. 2024 Jan 9;25(1):bbad485. doi: 10.1093/bib/bbad485 (PMC10782803; doi:10.1093/bib/bbad485)
Supplement: tutorial_methylClass_bbad485 [file tutorial_methylclass_bbad485.docx]

Tutorial for R package methylClass

Yu Liu

10/12/2022

## Introduction

DNA methylation profiling is emerging as a useful tool to increase the accuracy of cancer diagnosis *[1, 2]*. However, a comprehensive R package specially for it is still lacking. Hence, we developed the R package *methylClass* to handle the issues of methylation-based classification. Within it, we provide the eSVM (ensemble-based support vector machine) model able to achieve better accuracy in methylation data classification than the popular random forest (RF) model and also overcome the time-consuming problem of traditional SVM. In addition, some novel feature selection methods, such as *SCMER*, are included in the package to improve the classification *[3]*. Furthermore, in view that methylation data can be converted to other types of omics, such as copy number variation (CNV) data, we also provide several functions for multi-omics study.

## Package installation

The Code of *methylClass* is freely available at <https://github.com/yuabrahamliu/methylClass>.

The following commands can be used to install this R package.

library(devtools)

install_github('yuabrahamliu/methylClass')

## Data preparation

To demonstrate the functions of *methylClass*, we use a dataset that accompanies the package in this tutorial. It is a subset of the GSE109381 neural tumor dataset *[1]*. We only use its largest 7 sample classes, and the data includes a matrix with 740 rows and 12000 columns. Each row represents a sample, and each column represents a DNA methylation probe from the Infinium EPIC BeadChip platform. The 12000 columns here are the top 12000 most variable probes in the dataset, and the values recorded in this matrix are the DNA methylation beta values detected from the samples, which are brain tumor tissues from donors. The 740 samples cover 7 classes: 1) GBM, RTK II (Glioblastoma, RTK II subtype, 143 samples), 2) MB, G4 (Medulloblastoma, G4 subtype, 138 samples), 3) LGG, PA PF (Low-grade glioma, pilocytic astrocytoma in posterior fossa, 114 samples), 4) EPN, PF A (Ependymoma, posterior fossa ependymoma group A, 91 samples), 5) MNG (Meningioma, 90 samples), 6) MB, SHH CHL AD (Medulloblastoma, SHH subtype, child and adolescent, 84 samples), and O IDH (Oligodendroglioma IDH-mutant, 80 samples). The row names of the matrix are the sample IDs, while the column names are the DNA methylation probe IDs.

In addition to this matrix, a factor is also with this dataset. It records the class labels for the 740 samples. Each element is a label for one sample, and the element order is the same as the sample order in the beta value matrix.

Now, attach *methylClass* to the R session and get the example data.

library(methylClass)

betas <- system.file('extdata', 'testbetas.rds', package = 'methylClass')
betas <- readRDS(betas)

labels <- system.file('extdata', 'testlabels.rds', package = 'methylClass')
labels <- readRDS(labels)

The beginning parts of the beta value matrix and the labels are shown below.

betas[1:6, 1:6]
#> cg03646096 cg14689219 cg24545961 cg16765600 cg25718467
#> 6969568005_R02C01 0.3330177 0.0000643437 0.05823423 0.9861613 0.018482062
#> 6929689168_R02C02 0.9587832 0.0398763312 0.03262521 0.9768717 0.020495526
#> 6969568005_R04C01 0.6112368 0.0068696369 0.04922174 0.9616918 0.009739827
#> 6969568005_R05C01 0.9490475 0.0156952427 0.13558987 0.9899455 0.037586383
#> 6969568005_R06C01 0.5458699 0.0199477797 0.06596685 0.9698546 0.016476910
#> 6929689168_R03C02 0.9238038 0.0611110556 0.05906609 0.9551898 0.019658186
#> cg23714773
#> 6969568005_R02C01 0.9592555
#> 6929689168_R02C02 0.9404314
#> 6969568005_R04C01 0.9124694
#> 6969568005_R05C01 0.9466208
#> 6969568005_R06C01 0.9450230
#> 6929689168_R03C02 0.9265127

head(labels)
#> 6969568005_R02C01 6929689168_R02C02 6969568005_R04C01 6969568005_R05C01
#> LGG, PA PF LGG, PA PF LGG, PA PF LGG, PA PF
#> 6969568005_R06C01 6929689168_R03C02
#> LGG, PA PF LGG, PA PF
#> 7 Levels: GBM, RTK II < MB, G4 < LGG, PA PF < EPN, PF A < ... < O IDH

## Cross-validation

We will use these data to train 2 classifiers to distinguish different sample classes. One is an RF (random forest) classifier, the other is an eSVM (ensemble-based support vector machine) classifier, and we use a normal 5-fold cross-validation (CV) method to evaluate their performances. This can be done via the functions maincv and maincalibration in the package.

We first use maincv to train the raw models in a 5-fold CV and then use maincalibration to calibrate the raw model results and improve the performance.

For the RF CV models, we provide the 740 by 12000 betas matrix to maincv via its parameter betas.. and transfer the labels via the parameter y... We set another parameter subset.CpGs as 10000, so for each CV loop, the top 10000 most variable probes will be selected, and the RF model for this loop will be trained. The default values of the parameters n.cv.folds is 5 and normalcv is FALSE, so that a 5 by 5 nested CV will be performed, but here we change normalcv to TRUE to save time so that a normal 5 fold CV will be performed with only the 5 outer loops of the 5 by 5 nested CV. Then, we set out.path as “RFCV” so that a folder named “RFCV” will be created in the current working directory to save the result files of this function, and another parameter out.fname has a default value as “CVfold” so that all the files saved will have a prefix “CVfold” in their names. Finally, set the parameter method as “RF” so that RF models will be trained.

maincv(y.. = labels,
 betas.. = betas,
 subset.CpGs = 10000,
 n.cv.folds = 5,
 normalcv = TRUE,
 out.path = 'RFCV',
 out.fname = 'CVfold',
 method = 'RF',
 seed = 1234)

After this raw model training, we use the function maincalibration to calibrate the raw results. We transfer the labels to this function also via the parameter y.., and because maincalibration depends on the results of maincv, the folder with the maincv result files should be transferred to it via the parameter load.path, and the file name prefix should be transferred via load.fname. Actually, load.path and load.fname here correspond to out.path and out.fname in maincv. Then, because maincv trained the models on normal CV loops, not the nested ones, here, the normalcv parameter also needs to be set as TRUE. Finally, tell the function the models are RF models via setting the parameter algorithm as “RF”.

RFres <- maincalibration(y.. = labels,
 load.path = 'RFCV',
 load.fname = 'CVfold',
 normalcv = TRUE,
 algorithm = 'RF',
 setseed = 1234)

Then, the result RFres is a matrix with the performance of the RF model in the 5-fold CV for the raw model and all the calibrated models.

RFres
#> misc.error auc.HandTill brier mlogloss
#> rf 0 1 0.005541276 0.03359125
#> rf_LR 0 1 3.471967e-09 1.537957e-06
#> rf_FLR 0 1 0.0001563735 0.006696746
#> rf_MR 0 1 0.0004247702 0.006977818

You can see the mlogloss (cross-entropy loss) of the raw RF model (rf) is around 0.03359, while all the 3 calibration methods, i.e., LR (logistic regression), FLR (Firth’s regression) and MR (ridge regression), can reduce this loss and the best results are from LR, it has a loss of 1.538e-6.

The same things can be done to construct eSVM models for the 5-fold CV. Just note to change the parameters method and algorithm to “eSVM” and also set another folder name to save the maincv results for eSVM.

maincv(y.. = labels,
 betas.. = betas,
 subset.CpGs = 10000,
 n.cv.folds = 5,
 normalcv = TRUE,
 out.path = 'eSVMCV',
 out.fname = 'CVfold',
 method = 'eSVM',
 seed = 1234)

eSVMres <- maincalibration(y.. = labels,
 load.path = 'eSVMCV',
 load.fname = 'CVfold',
 normalcv = TRUE,
 algorithm = 'eSVM',
 setseed = 1234)

We can see the result of eSVM via the returned eSVMres.

eSVMres
#> misc.error auc.HandTill brier mlogloss
#> esvm 0 1 0.005083419 0.05225512
#> esvm_LR 0 1 1.478146e-14 3.326961e-09
#> esvm_FLR 0 1 5.113176e-05 0.006243303
#> esvm_MR 0 1 7.96243e-05 0.003659271

Here, we use the beta values in the data matrix to train the models, and for methylation data, it is also suggested to convert these betas to M values and try to train models on them to see if there is any large difference between the model performance of beta values and M values.

For the beta value model here, we evaluate the performance using the 5-fold CV, but actually, the parameter normalcv can also be set as FALSE, and then a 5 by 5 nested CV will be used to evaluate the model. It will take more time than the normal 5-fold CV, but it can prevent the problem of over-fitting for the calibration step more effectively.

Another parameter that needs to be noted is subset.CpGs. Here we set it as 10000 so that the top 10000 most variable probes are used to train the models, and any numeric number transferred to it will be explained as the number of top variable probes needed to construct the model. While another 2 types of features can also be selected via setting subset.CpGs as the string “SCMER” or “limma” so that the method *SCMER* will be used to select the features able to preserve the manifold structure of the original data *[3]*, while *limma* will be used to select the top differential probes between the sample classes *[4]*. Then, these probes can be used to train the models instead of the top variable ones. More details can be obtained from the help documents of the function.

For the RF and eSVM models trained here, if more details are needed for each CV loop, they can be found in the saved files generated by maincv and maincalibration, such as the prediction score matrix of testing samples, the trained model object, etc. If you do not need to know these details, these files can be deleted after getting the final evaluation matrices of RFres and eSVMres.

## Model construction

From RFres and eSVMres, we find eSVM performs better than RF during the 5-fold CV, so we next choose it to train a final model on the whole dataset without a training/testing set division. Then, this model can be used on external datasets to predict the unknown sample labels.

This is fulfilled via the function maintrain.

mods <- maintrain(y.. = labels,
 betas.. = betas,
 subset.CpGs = 10000,
 seed = 1234,
 method = 'eSVM',
 calibrationmethod = c('LR', 'FLR', 'MR'))

We set the parameter calibrationmethod with the vector c(‘LR’, ‘FLR’, ‘MR’) so that all of the 3 calibration methods will be used and the 3 calibrated models, as well as the raw model, will be returned with the result mods.

summary(mods)
#> Length Class Mode
#> mod 3 -none- list
#> rawscores 5180 -none- numeric
#> platt.calfits 7 -none- list
#> probs.lr 5180 -none- numeric
#> platt.brglm.calfits 7 -none- list
#> probs.flr 5180 -none- numeric
#> glmnet.calfit 12 cv.glmnet list
#> probs.mr 5180 -none- numeric

You can see mods contains 8 slots, and the one named ‘mod’ is the raw model trained from the whole dataset, while the ones named ‘platt.calfits’, ‘platt.brglm.calfits’, and ‘glmnet.calfit’ are the 3 calibrated models of ‘LR’, ‘FLR’, and ‘MR’. Other slots are the prediction scores of the models on the whole dataset.

Then, another function, mainpredict, can be used, and the different slots of mods and the external data you want to use to predict their sample labels can be transferred to this function to get the labels. More details can be found in the help document for mainpredict.

This package also contains other functions, such as multi-omics data classification and visualization (maincv and mainJvisR), DBSCAN clustering (function clustering), etc. We will not cover them in this tutorial to make it more simplified, and their details can be found in the help documents.

## References

1. Capper, D., Jones, D.T.W., Sill, M., Hovestadt, V., Schrimpf, D., Sturm, D., Koelsche, C., Sahm, F., Chavez, L., Reuss, D.E., et al. (2018). DNA methylation-based classification of central nervous system tumours. Nature 555, 469-474.
2. Koelsche, C., Schrimpf, D., Stichel, D., Sill, M., Sahm, F., Reuss, D.E., Blattner, M., Worst, B., Heilig, C.E., Beck, K., et al. (2021). Sarcoma classification by DNA methylation profiling. Nature Communications 12, 498.
3. Liang, S., Mohanty, V., Dou, J., Miao, Q., Huang, Y., Müftüoğlu, M., Ding, L., Peng, W., and Chen, K. (2021). Single-cell manifold-preserving feature selection for detecting rare cell populations. Nature Computational Science 1, 374-384.
4. Ritchie, M.E., Phipson, B., Wu, D., Hu, Y., Law, C.W., Shi, W., and Smyth, G.K. (2015). limma powers differential expression analyses for RNA-sequencing and microarray studies. Nucleic Acids Res 43, e47.
